# Electroacupuncture regulates Rab5a‐mediating NGF transduction to improve learning and memory ability in the early stage of AD mice

**DOI:** 10.1111/cns.14743

**Published:** 2024-05-23

**Authors:** Jianhong Li, Minguang Yang, Yaling Dai, Xiaoqin Guo, Yanyi Ding, Xiaoling Li, Shenghang Zhang, Wenshan Xu, Lidian Chen, Jing Tao, Weilin Liu

**Affiliations:** ^1^ The Institute of Rehabilitation Industry Fujian University of Traditional Chinese Medicine Fuzhou China; ^2^ Fujian Key Laboratory of Aptamers Technology 900TH hospital of Joint Logistics Support Force Fuzhou China; ^3^ National‐Local Joint Engineering Research Center of Rehabilitation Medicine Technology Fujian University of Traditional Chinese Medicine Fuzhou China; ^4^ Provincial and Ministerial Co‐founded Collaborative Innovation Center of Rehabilitation Technology Fujian University of Traditional Chinese Medicine Fuzhou China; ^5^ Fujian Key Laboratory of Cognitive Rehabilitation Affiliated Rehabilitation Hospital of Fujian University of Traditional Chinese Medicine Fuzhou China; ^6^ Traditional Chinese Medicine Rehabilitation Research Center of State Administration of Traditional Chinese Medicine Fujian University of Traditional Chinese Medicine Fuzhou China

**Keywords:** Alzheimer's disease, cholinergic neural circuit, electroacupuncture, learning and memory, Rab5a

## Abstract

**Aims:**

Nerve growth factor (NGF) loss is a potential factor for the degeneration of basal forebrain cholinergic neurons (BFCNs) in Alzheimer's disease (AD), and Rab5a is a key regulatory molecule of NGF signaling transduction. Here, we investigated the changes of Rab5a in 5 × FAD mice and further explored the mechanism of Electroacupuncture (EA) treatment in improving cognition in the early stage of AD.

**Methods:**

The total Rab5a and Rab5a‐GTP in 5‐month‐old 5 × FAD mice and wild‐type mice were detected using WB and IP technologies. 5 × FAD mice were treated with EA at the Bai hui (DU20) and Shen ting (DU24) acupoints for 4 weeks and CRE/LOXP technology was used to confirm the role of Rab5a in AD mediated by EA stimulation. The Novel Object Recognition and Morris water maze tests were used to evaluate the cognitive function of 5 × FAD mice. The Nissl, immunohistochemistry, and Thioflavin S staining were used to observe pathological morphological changes in the basal forebrain circuit. The Golgi staining was used to investigate the synaptic plasticity of the basal forebrain circuit and WB technology was used to detect the expression levels of cholinergic‐related and NGF signal‐related proteins.

**Results:**

The total Rab5a was unaltered, but Rab5a‐GTP increased and the rab5a‐positive early endosomes appeared enlarged in the hippocampus of 5 × FAD mice. Notably, EA reduced Rab5a‐GTP in the hippocampus in the early stage of 5 × FAD mice. EA could improve object recognition memory and spatial learning memory by reducing Rab5a activity in the early stage of 5 × FAD mice. Moreover, EA could reduce Rab5a activity to increase NGF transduction and increase the levels of phosphorylated TrkA, AKT, and ERK in the basal forebrain and hippocampus, and increase the expression of cholinergic‐related proteins, such as ChAT, vAchT, ChT1, m1AchR, and m2AchR in the basal forebrain and ChAT, m1AchR, and m2AchR in the hippocampus, improving synaptic plasticity in the basal forebrain hippocampal circuit in the early stage of 5 × FAD mice.

**Conclusions:**

Rab5a hyperactivation is an early pathological manifestation of 5 × FAD mice. EA could suppress Rab5a‐GTP to promote the transduction of NGF signaling, and enhance the synaptic plasticity of the basal forebrain hippocampal circuit improving cognitive impairment in the early stage of 5 × FAD mice.

## Graphical abstract

A diagram illustrating the mechanism of basal forebrain hippocampal circuit plasticity enhancement by EA treatment. Overexpression of Rab5a‐GTP in 5 × FAD mice promotes early endosome fusion and facilitates NGF/TrkA complexes containing endosome degradation, resulting in NGF signal loss and a decrease in basal forebrain hippocampal circuit plasticity. EA treatment reduces the level of Rab5a‐GTP in 5 × FAD mice, increasing the NGF signal and basal forebrain hippocampal circuit plasticity in the early stage of AD.

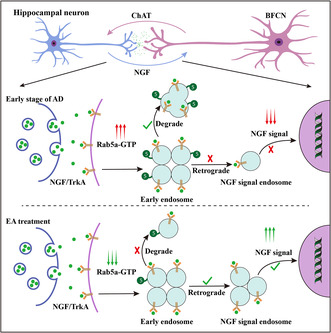

## 1 INTRODUCTION

Alzheimer's disease (AD) is a neurodegenerative disease characterized by progressive cognitive dysfunctions such as memory loss, disorientation, and language difficulty. The cholinergic hypothesis is the mainstream hypothesis of AD and states that patients suffering from AD experience cognitive dysfunction due to the specific vulnerability of BFCNs.^1^, ^2^ Thus far, Acetylcholinesterase inhibitors (AchEi) targeting the cholinergic system are the most commonly used drugs against AD in clinical treatments. However, these anti‐AD medications are only capable of temporarily alleviating the symptoms of cognitive impairment and come with several side effects, such as nausea, diarrhea, and abdominal pain.^3^ The amyloid cascade hypothesis posits that Amyloid β peptide (Aβ) aggregation is the initial event of Alzheimer's disease, followed by synapse loss and neuronal death leading to cognitive impairment.^4^ We suspected that Aβ neurotoxicity might not be the proximate cause of neuronal dysfunction in the basal forebrain, since in a previous study we did not detect Aβ plaques in APP/PS1 mice, even at 13 months of age, when synaptic loss and neuronal degeneration typically become apparent.^5^ Therefore, reviewing the various hypotheses of AD pathology and redefining possible causal events could aid in the discovery of a novel mechanism for AD pathology.

Given that BFCNs have an extraordinary architecture, with long axons and complex dendrites that help exert their function, it is unsurprising that safe and effective endocytic pathways are critical for maintaining the structure and normal function of BFCNs.^6^ NGF is a type of endogenous neurotrophic factor involved in regulating the growth, maintenance, and survival of BFCNs by binding with the tyrosine receptor kinase A (TrkA).^7^, ^8^, ^9^ Following endocytosis, NGF/TrkA signaling complexes are trafficked to early endosomes. These are also called “signaling endosomes,” which are retrogradely transported to the cell bodies of the neurons to activate NGF/TrkA‐inducible gene expression and promote neurite outgrowth.^10^, ^11^ Genome‐wide association studies (GWAS) and genome‐wide association meta‐analyses (GWAMAs) have recently identified a significant number of new AD risk genes encoding products that predominantly function in endocytic trafficking and signal transduction, such as Bridging Integrator 1 (BIN1), Phosphatidylinositol Binding Clathrin Assembly Protein (PICALM), CD2 Associated Protein (CD2AP), and Sortilin‐related receptor (SORL1).^12^, ^13^ As such, NGF signal loss in the endosomal pathway could lead to basal forebrain neuronal dysfunction.

The small guanosine triphosphate (GTP)ase Rab5 regulates NGF signaling by controlling early endosome fusion. Like all GTP‐binding proteins, Rab5 activity is regulated by guanine nucleotide exchange factors (GEF) and GTPase activating proteins (GAP), cycling between an inactivate GDP‐bound and active GTP‐bound form, regulating endocytosis, intracellular vesicular trafficking, and NGF signaling transduction.^14^ Endocytic dysfunction is an early pathological event in AD and the Rab5‐positive early endosome enlargement is a unique feature of AD.^15^ Additionally, morphological alterations of Rab5‐positive early endosomes appear earlier than the pathologies of neurofibrillary tangle and glial activation.^16^ Several studies have found that Rab5 protein levels in the basal forebrain, hippocampus, and frontal cortex were elevated in mild cognitive impairment (MCI) and AD patients.^17^, ^18^ Rab5 overexpression was also observed in AD mouse models and human induced pluripotent stem cells (iPSC) lines carrying autosomal dominant Familial AD (fAD) amyloid precursor protein (APP) and Presenilin 1 (PS1) mutations.^19^, ^20^ However, Ts65Dn mice with increased APP gene dose only exhibited hyperactivated Rab5 and larger early endosomes, but not overexpression of total Rab5.^21^

In this study, we first examined total Rab5a and Rab5a‐GTP expression levels to determine the pathological changes in 5 × FAD mice and further explore the possible molecular mechanism by which Rab5a affects BFCNs function in the early stage of AD. Studies found that EA stimulation could increase NGF signaling to enhance levels of the Choline acetyltransferase (ChAT) enzyme in cholinergic neurons of the medial septum in AD model rats.^22^ In a previous study, we found that EA stimulation could increase the levels of cholinergic‐related proteins and enhance functional connectivity of the basal forebrain hippocampal cholinergic neural circuit.^23^ Here, we used EA stimulation to explore the role of Rab5a and further elucidate the molecular mechanism of EA rescuing BFCN function by regulating Rab5a in the early stage of AD.

## 2 MATERIALS AND METHODS

### 2.1 Animals and ethics

**FIGURE 1:**
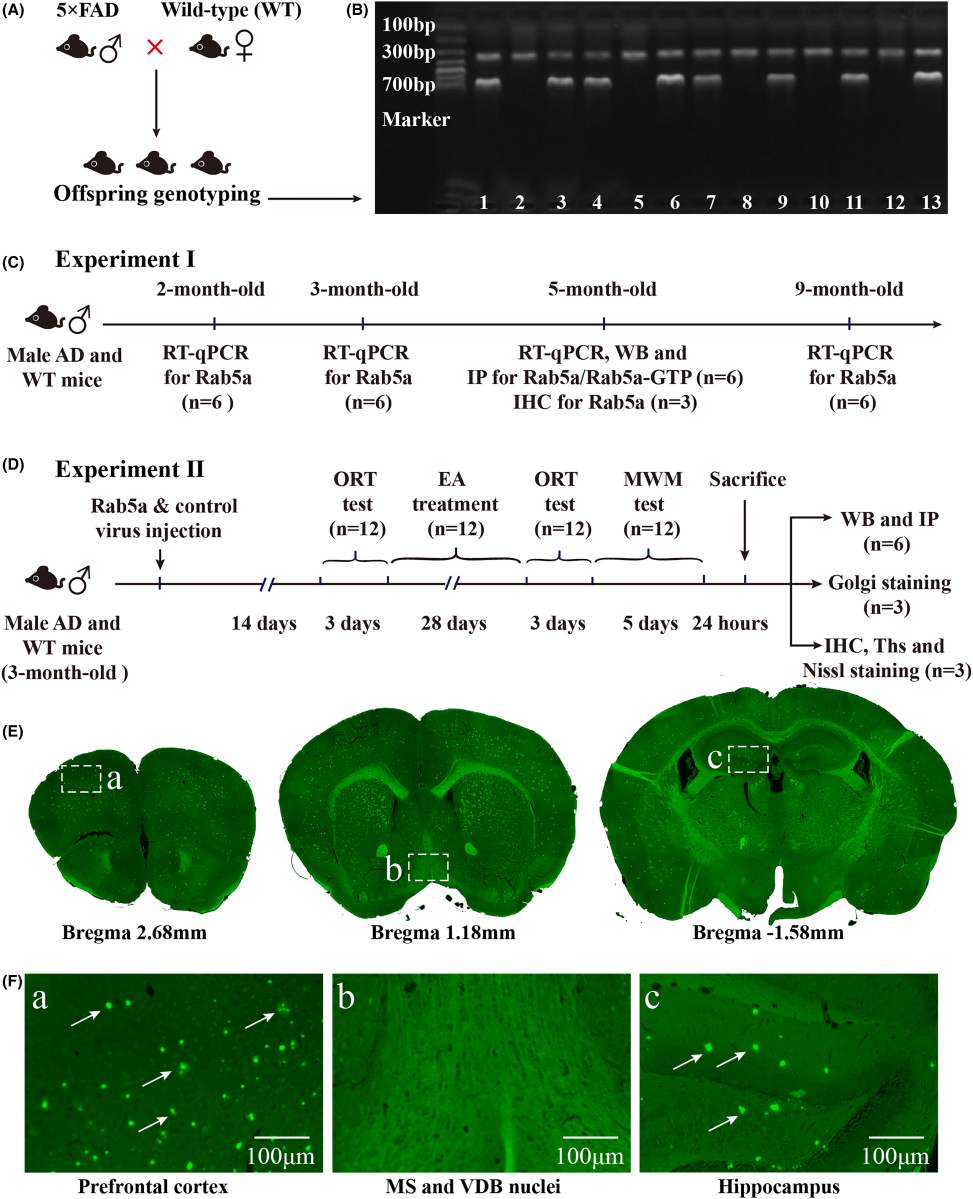
Breeding strategy and flowcharts of the experimental procedure. (A) Breeding strategy where 5 × FAD males were crossed with C57BL/6 wild‐type females to generate offsprings. (B) Representative images of PCR results used for genetic identification, the PCR product bands of APP (350 bp), PS1 (608 bp) and GAPDH (324 bp) were identified clearly. (C) Flowchart of the experiment I. (D) Flowchart of the experiment II. (E) Representative images of Aβ plaques detected in the brain regions of the prefrontal cortex, basal forebrain and hippocampus. (F) Partially enlarged view of figure E (100×). The Aβ plaques were detected in the prefrontal cortex(a) and hippocampus(c) (as shown by the arrows). APP, amyloid precursor protein; PS1, Presenilin 1.

Male 5 × FAD transgenic mice were purchased from Qianbi Biotechnology (stock #34848, Qianbi Biotechnology Co., Ltd., China). 5 × FAD mice co‐express five fAD mutations: APP with K670N/M671L (Swedish mutation), I716V (Florida mutation), V717I (London mutation), and PS1 with M146L and L286V mutations. The animals were fed and cultured in the Specific Pathogen Free (SPF) laboratory of the Experimental Animal Center of Fujian University of Traditional Chinese Medicine [license number: SYXK (Min) 2019–0007]. We produced a mice line by crossing male 5 × FAD mice with female C57BL/6 wild‐type mice (Figure 1A). The offspring genotyping was determined by polymerase chain reaction (PCR). The mice with positive APP (350 bp) and PS1 (608 bp) bands were identified as 5 × FAD mice, while only the GAPDH (324 bp) bands that showed positive were littermate wild‐type controls (Figure 1B). The mice were housed in cages (3–5 animals per cage) at 21–25°C under a 12 h light/12 h dark cycle with food and water ad libitum. The animal experimental study was carried out in compliance with the ethical regulations approved by the Animal Ethics Committee of Fujian University of Traditional Chinese Medicine (Fuzhou, Fujian province, China).

### 2.2 Experimental design and animal grouping

In experiment I (Figure 1C), the mRNA and protein expression levels of Rab5a in 5 × FAD mice and age‐matched wild‐type mice were analyzed at 2, 3, 5, and 9 months of age. 5 × FAD mice and wild‐type mice were randomly placed into the 2mo‐AD group, 2mo‐WT group, 3mo‐AD group, 3mo‐WT group, 5mo‐AD group, 5mo‐WT group, 9mo‐AD group, and 9mo‐WT group, with 6 animals per group. We used the results of experiment I to explore the underlying mechanism of EA stimulation responsible for improving the cognitive function of AD mice in experiment II (Figure 1D), in which 3‐months‐old 5 × FAD mice were randomly divided into 4 groups, with 12 animals per group: AD group, EA group, Rab5a‐EA group, and NC‐EA group. An additional 12 wild‐type mice were placed in the WT group.

### 2.3 Virus injection

After being anesthetized with 3% isoflurane (Ruiwode Lifescience Co., Ltd), the hair of each mouse's head was shaved. Next, the mouse was fixed in the stereoscopic locator (Ruiwode Lifescience Co., Ltd) and 1% isoflurane was used to maintain anesthesia. In experiment II, mice in the Rab5a‐EA group were transfected with Rab5a‐overexpression virus (consisting of rAAV‐chat‐cre‐2a‐EGFP‐WPRE‐pA and rAAV‐efla‐dio‐rab5a‐mcherry‐WPRE‐pA mixed in a 1:3 ratio, 1 × 10^12^ particles/ml, Wuhan Institute of Physics and Mathematics). Mice in the NC‐EA group were transfected with the control virus (rAAV‐chat‐cre‐2a‐EGFP‐WPRE‐pA, 1 × 10^12^ particles/ml). Using a 10 μL precision Hamilton micro‐syringe, a total of 400 nL viral solutions were injected into the MS and VDB nuclei of the basal forebrain (AP +1.10 mm, ML 0.00 mm, DV −4.80 mm; relative to the bregma). The virus was infused at a rate of 50 nL/min for 8 min and the micro‐syringe was left in place for an additional 5 min before being slowly withdrawn. Additional experiments were performed 2 weeks after surgery.

### 2.4 EA intervention

According to previous studies, EA stimulation was performed at the DU20 and DU24 acupoints. The DU20 acupoint is located at the intersection of the line linking the ear tips of the mice and the sagittal midline, and the DU24 acupoint is located in front of the junction of the sagittal midline and the suture. For EA stimulation, mice in the EA group, Rab5a‐EA group, and NC‐EA group (experiments II) were fixed on a homebuilt setup. The acupuncture needles (0.32 mm diameter; Huatuo, Suzhou Medical Appliance Factory) were inserted at a depth of 5 ~ mm into the DU20 and DU24 acupoints. After the needles were applied, the EA instrument (model: SDZ‐V, Suzhou Medical Appliance Factory) was connected with a frequency of sparse and dense waves of 1/20 Hz, a voltage of 2 V, for 30 min, once a day, 5 times a week, for 4 weeks.

### 2.5 New object recognition (NOR)

The NOR test is used to evaluate the ability of each mouse to recognize a novel object based on their tendency to explore a novel object instead of a known old object. The experimental apparatus consisted of a white rectangular open field (50 × 50 × 50 cm). Object “A” was the same as object “a”, which were considered familiar objects. There was also an unfamiliar object “B”. The NOR experiment consisted of 3 phases: the adaptation phase, the familiarization phase, and the test phase. The day before testing, experimental mice were placed in an open field for 5 min to adapt to their environment. In the familiarization phase, two identical objects (A and a) were positioned in two adjacent corners of the open field, and the mice were placed into the open field to freely explore for 10 min and then returned to the cages. In the test phase, 1 and 24 h after the familiarization phase, object “a” was replaced with the novel object “B”, and the mice were placed into the open field, allowed to freely explore for 5 min, and returned to the cages. The experimental apparatus was cleaned with 75% alcohol to eliminate the odor of the previous mouse. Time spent exploring each object was recorded by a camera installed directly above the apparatus. The discrimination index (DI) was calculated: DI = Time exploring B/(Time exploring A + Time exploring B) × 100%.

### 2.6 Morris water maze (MWM)

The MWM test was used to evaluate the spatial learning and memory ability of each mouse. This experimental apparatus consisted of a white circular pool (diameter 120 cm, height 50 cm). The Morris water maze was filled with water to a height of 30 cm (24 ± 1°C) and divided into four quadrants of equal size, numbered from 1 to 4. An escape platform (7.5 cm in diameter) was placed 2 cm below the water line in the center of the 3rd quadrant and remained in the same position. The MWM experiment consisted of 2 phases: the positioning navigation phase and the space exploration test phase. During the positioning navigation phase, each mouse was placed in the water at random start locations: the 1st quadrant, the 2nd quadrant, the 3rd quadrant, and the 4th quadrant (4 trials a day, lasting 4 days). If the mice found the escape platform within 60 s, the actual escape latency was recorded. Otherwise, the escape latency were recorded as 60 s and the mice were guided to the platform by the experimenter for 10 s. After the positioning navigation phase, the mice conducted a space exploration test. In this phase, the escape platform was removed and each mouse was placed into the water from the 1st quadrant and allowed to freely explore for 60 s. The number of platform crossing times and the amount of time spent in the target quadrant were recorded by the camera installed directly above the apparatus.

### 2.7 Real‐Time Quantitative PCR (RT‐qPCR)

The expression levels of target genes were calculated by relative quantification using the 2−∆∆CT method. Total RNA was extracted by the Trizol method, according to the manufacturer's instructions. Reverse transcription was performed using the HiScript II Q Select RT SuperMix for the qPCR kit (Vazyme Biotech Co., Ltd). Real‐time PCR was performed with a ChamQ Universal SYBR qPCR Master Mix (Vazyme Biotech Co., Ltd.). The primer sequences of Rab5a (Gene ID: 271457) are listed below: GCTAATCGAGGAGCAACAAGAC (forward); CCAGGCTTGATTTGCCAACAG (reverse). For house‐keeping gene GAPDH (Gene ID: 14433), the primer sequences were TGGAAAGCTGTGGCGTGATG (forward) and TACTTGGCAGGTTTCTCCAGG (reverse).

### 2.8 Thioflavin‐S (ThS) and Nissl staining

Anesthetized mice (pentobarbital sodium [0.05 g/kg i.p.]) were perfused with saline to wash out the blood and were then perfused with 4% paraformaldehyde to fix the brain tissue. The brain tissue was embedded in paraffin and 5 μm coronal slices of brain tissue samples were prepared for pathological staining. For ThS staining, the brain sections were routinely deparaffinized, hydrated, placed in 0.3% ThS solution (Thioflavin S, Sigma, T1892) for 8 min, and differentiated in 50% alcohol for 3 × 5 min. For Nissl staining, the brain sections were routinely deparaffinized and hydrated, and the sections were incubated for 1 h at 56°C with A reagent (Nissl Staining Solution (Cresyl Violet), Solarbio, G3410). The brain sections were then incubated in B reagent for 2–3 min. Finally, the brain sections were sealed with neutral resin and photographed under a microscope.

### 2.9 Immunohistochemistry (IHC) staining

The 5 μm coronal slices of the brain tissues were prepared for Rab5a and ChAT IHC staining. The brain sections were routinely deparaffinized and hydrated and antigen repair was performed by placing the brain sections in the boiled citric acid buffer for 1–2 h, after which they were washed in PBS for 5 min. For Rab5a staining, the operating steps were followed strictly according to the kit instructions (Maixin, KIT‐9720). The primary antibodies Rab5a were incubated at 4°C overnight (CST, E6N8S, 1:100). For ChAT staining, the brain sections were then incubated with 0.3% H_2_O_2_ at room temperature for 10 min, after which they were washed in PBS for 10 min and incubated in 3% bovine serum albumin (BSA) at room temperature for another 30 min. They were then incubated in ChAT primary antibodies (Sigma, AB144P, 1:100) for two nights at 4°C. Following incubation, the brain sections were washed in PBS for 10 min and incubated with secondary antibody (Vector Laboratories, PK4005, 1:100) for 2 h at room temperature, washed in PBS for 5 min, and incubated with ABC‐kit solution (Vector Laboratories, PK‐4005) for 2 h at room temperature. Next, the brain sections were washed in PBS for 10 min, stained in DAB staining solution for 3 min, and quickly washed in PBS. The nuclei were counterstained with hematoxylin for 2 min and the brain sections were sealed with neutral resin and photographed under a microscope.

### 2.10 Golgi staining

Golgi staining was performed using the FD Rapid Golgi Stain kit (PK401). The mice were euthanized and their brains were submerged in mixed Reagent A and Reagent B (1:1) for 2 weeks and were protected from light. The brain tissues were then incubated in Reagent C for 5 days. Next, the brain tissues were embedded in OCT and 100 μm frozen sections were cut. The brain slices were stained according to the manufacturer's instructions. The brain tissues were subsequently dehydrated using graded alcohol (50%, 70%, 90%, 95%, and 100%) and were made transparent with xylene and sealed with neutral resin. The morphology and number of dendritic spines in the hippocampal neurons were observed under a 600× microscope.

### 2.11 Western blotting (WB)

Brain homogenates were extracted with RIPA lysate for Western blot analysis. The protein concentrations were quantified using the BCA method to 5 μg/μL, and the protein lysates were separated using SDS‐PAGE methods. The proteins were transferred to the PVDF membrane (Merck Millipore) through a wet‐transfer protocol, and the membranes were blocked with 5% skimmed milk for 1 h at room temperature and washed in TBST for 3 × 5 min. The membranes were incubated with primary antibodies at 4°C: Rab5a (1:1000; 24 h, CST, E6N8S); Rabep1 (1:5000; 24 h, Abcam, ab176578); TrkA (1:300; 36 h, Abcam, ab216626); pTrkA (1:500; 36 h, Invitrogen, PA5‐37672); AKT (1:1000; 24 h, CST, #4691); pAKT (1:1000; 36 h, CST, #4060); ERK (1:5000; 24 h, Abcam, ab184699); pERK (1:2000; 24 h, CST, #4370); ChAT (1:5000; 24 h, Abcam, ab181023); AchE (1:5000; 36 h, Abcam, ab183591); vAchT (1:1000; 36 h, Sigma, sab4200559); ChT1 (1:5000; 24 h, Abcam, ab154186); m1AchR (1:1000; 36 h, boster, BA1543); m2AchR (1:5000; 24 h, Abcam, ab109226); GAPDH (1:5000; 24 h, Proteintech, 60,004–1‐1 g); and β‐actin (1:5000; 24 h, Proteintech, 66,009–1‐1 g). Following primary antibody incubation, the membranes were washed in TBST for 3 × 5 min and incubated with HRP‐conjugated secondary antibody (1:5000) for 1 h at room temperature. The protein bands were visualized and imaged by the Bio‐Rad ChemiDoc Imaging System.

### 2.12 Protein immunoprecipitation (IP)

Brain homogenates were extracted with NP‐40 lysate (Beyotime Biotechnology) for protein immunoprecipitation. The protein concentrations were quantified using the BCA method to 1 μg/μL. Volumes containing 400 μg of protein were incubated with 2 μL of anti‐Rabep1 antibody (Abcam, ab176578) or rabbit IgG (Beyotime, A7058) for 24 h at 4°C in 400 μL of immunoprecipitation buffer. Subsequently, 40 μL of protein agarose A/G beads (Beyotime Biotechnology) was added to each sample for an additional 24 h at 4°C. Samples were centrifuged at 3000 rpm for 5 min at 4°C, and the immune complexes were collected. Rabep1 and Rabep1 binding protein Rab5a were detected using WB, as previously described.

### 2.13 Fluorescence microscopy verification of viral expression

Anesthetized mice (pentobarbital sodium [0.05 g/kg i.p.]) were perfused with saline to wash out the blood and were then perfused with 4% paraformaldehyde to fix the brain tissue. The brain tissue was embedded in OCT and 5 μm frozen sections were cut for fluorescence microscope imaging.

### 2.14 Statistical analysis

The data were analyzed using SPSS23.0 software and the results were expressed as the mean ± SEM. The normality of data distribution was tested using the Shapiro–Wilk test. In experiment I, we applied an independent sample *t‐*test to observe the difference between the 5 × FAD and age‐matched wild‐type mice. In experiment II, the escape latency data in the MWM test were analyzed by repeated measurements of variance analysis, and other data were analyzed by one‐way ANOVA. The intergroup comparisons of the data with equal variances were performed using the LSD method, while the Games Howell method was used for data with unequal variances. The difference was considered significant when *P* < 0.05.

## 3 RESULTS

### 3.1 Rab5a protein was hyperactivated and Rab5a‐positive early endosomes enlarged in 5 × FAD mice

**FIGURE 2:**
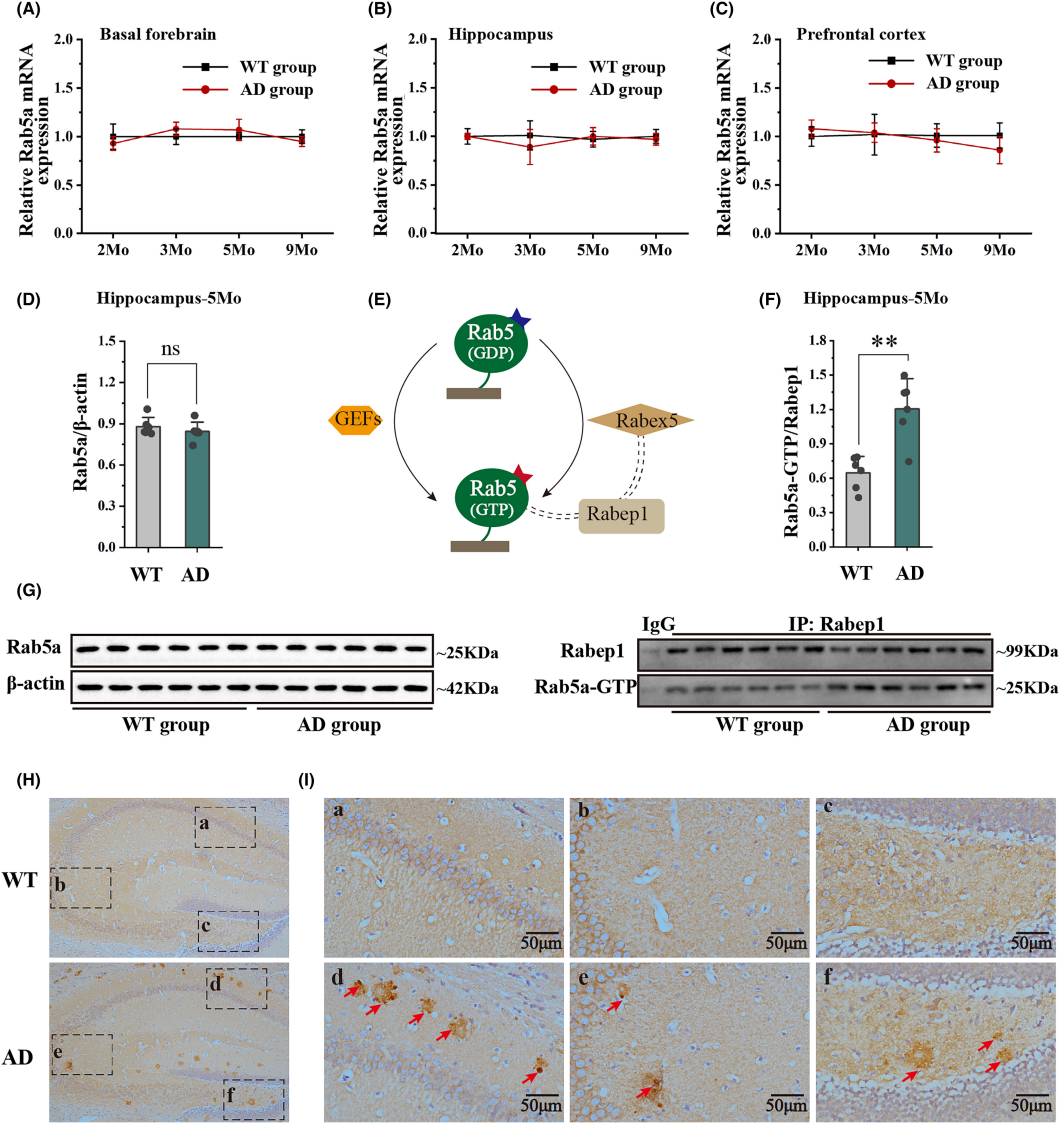
The expression levels of Rab5a and the morphology of rab5a‐positive early endosomes. (A‐C) The relative mRNA levels of the Rab5a gene in the basal forebrain, hippocampus, and prefrontal cortex in 2‐, 3‐, 5‐ and 9‐month‐old 5 × FAD mice and age‐matched wild‐type mice (*n* = 6 for each group). (D) Protein levels of the Rab5a gene in the hippocampus in 5‐month‐old 5 × FAD mice and age‐matched wild‐type mice (*n* = 6 for each group). (E) The schematic diagram of Rabep1 interaction with Rab5a‐GTP, but not Rab5a‐GDP. (F) Binding rate of Rab5a‐GTP to Rabep1 in the hippocampus of 5‐month‐old 5 × FAD mice and age‐matched wild‐type mice (*n* = 6 for each group, *P < 0.05 vs WT group). (G) Greyscale image of proteins. (H) Rab5a‐positive early endosomes in the hippocampus in 5‐month‐old wild‐type mice and 5 × FAD mice. (I) Partially enlarged view of figure H (200×), no enlarged Rab5a‐positive early endosomes were detected in CA1 (a), CA3 (b), DG (c) in 5‐month‐old wild‐type mice, and the rab5a‐positive early endosomes appear enlarged in CA1 (d), CA3 (e), DG (f) in 5‐month‐old 5 × FAD mice (as shown by the arrows).

In a previous study, we detected No Aβ deposition in the basal forebrain in 6‐, 8‐, and 13‐month‐old APP/PS1 mice.^5^ Therefore, we evaluated the distribution of Aβ plaques in 5 × FAD mice. As expected, the results of ThS staining showed numerous Aβ plaques were widely distributed throughout the prefrontal cortex, hippocampus, and entorhinal cortex. However, no Aβ plaques were detected in the basal forebrain in 5‐month‐old 5 × FAD mice (Figure 1E,F). This suggested that the dysfunction of BFCNs in the basal forebrain could be related to the impairment of Rab5‐positive early endosomes. We measured both Rab5a mRNA and protein levels in 5 × FAD mice. We first used RT‐qPCR to measure mRNA levels in cognitive‐related brain regions from 2‐ to 9‐month‐old 5 × FAD mice and compared the measurements with their age‐matched wild‐type mice. 5 × FAD mice showed no significant changes in Rab5a mRNA levels in the basal forebrain, hippocampus, and prefrontal cortex (Figure 2A–C). To assess if Rab5a expression also remained unchanged in 5 × FAD, we used WB to measure Rab5a proteins in the hippocampus of 5‐month‐old 5 × FAD mice. The results confirmed that Rab5a protein levels remained unchanged in 5 × FAD mice compared to age‐matched wild‐type mice (Figure 2D,G). Next, the activation levels of Rab5a (Rab5a‐GTP) were determined by IP Western blot analysis. Rab GTPase‐binding Effector Protein 1 (Rabep1) is an effector of Rab5a, and we used the Rabep1 antibody to purify and detect Rab5a‐GTP protein expression levels (Figure 2E). A higher binding rate indicates higher levels of Rab5a activation, and the results showed that the binding rate of Rab5a to Rabep1 increased in the hippocampus of 5‐month‐old 5 × FAD mice compared to age‐matched wild‐type mice (Figure 2F,G). Simultaneously, the results of IHC staining showed that the Rab5‐positive early endosomes appear enlarged in the hippocampus of 5‐month‐old 5 × FAD mice, including hippocampal CA1, CA3 and DG subfields (Figure 2H,I).

### 3.2 Rab5a hyperactivation was induced using a recombinant adeno‐associated virus

**FIGURE 3:**
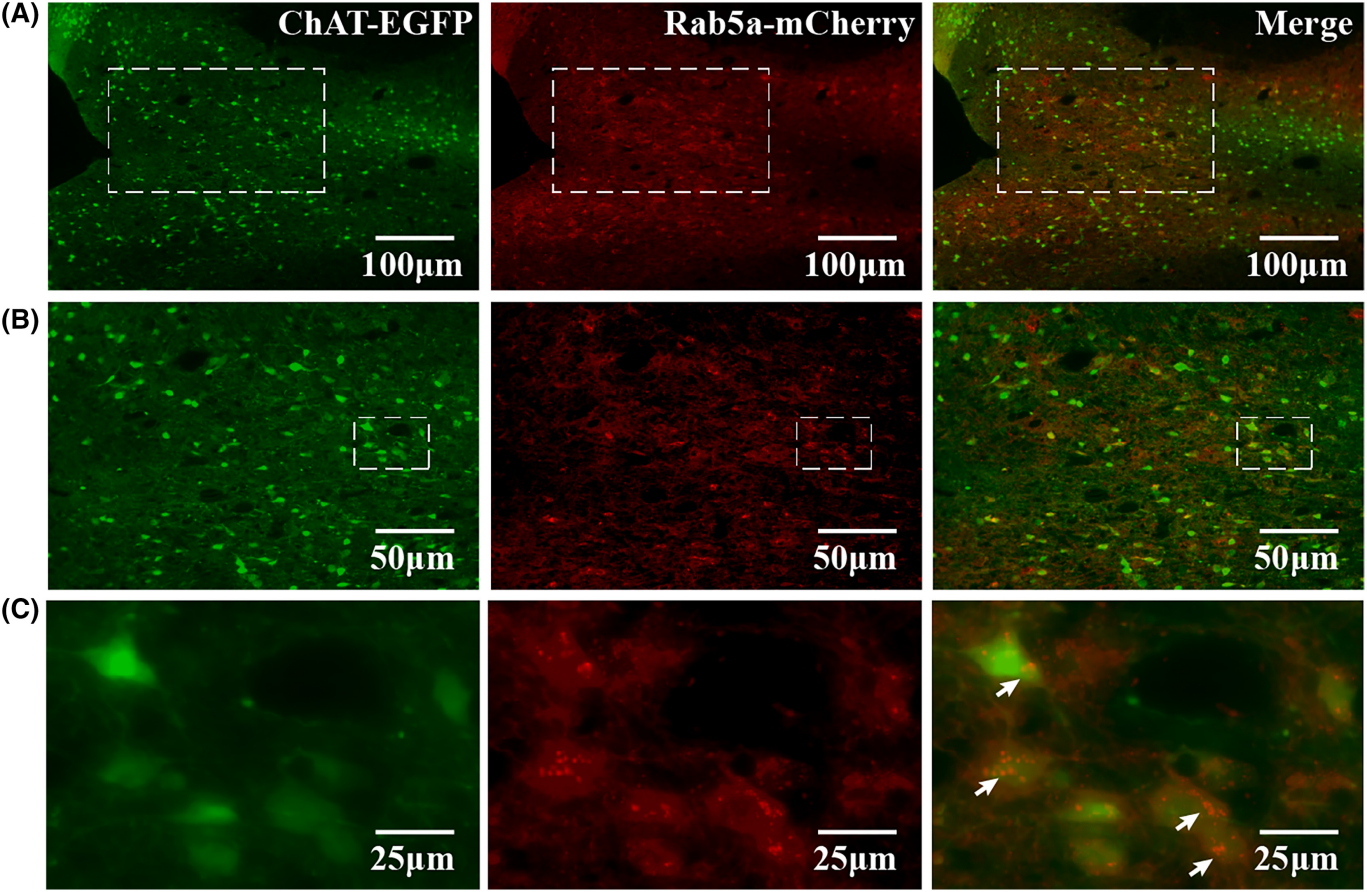
Validation of the expression of Rab5a. (A) AAV virus‐transfected Rab5a was successfully expressed on cholinergic neurons in the MS and VDB nuclei of the basal forebrain (100×). (B) Partially enlarged view of figure A (200×), which shows that the Rab5a‐mCherry was colocalized with ChAT‐EGFP. (C) Partially enlarged view of figure B (400×), which shows enlarged Rab5a‐positive early endosomes (red punctates, as shown by the arrows). MS, medial septal; VDB, vertical limb of the diagonal band; ChAT, choline acetyltransferase.

A previous study found that transgenic mice selectively expressing the Rab5a human isoform in neurons caused Rab5a hyperactivation and early endosome dysfunction.^24^ In this study, the Rab5a protein was overexpressed in the basal forebrain using a recombinant adeno‐associated virus. Fluorescent imaging showed Rab5a‐mCherry colocalized with ChAT‐eGFP in the nuclei of the medial septal (MS) and vertical limb of the diagonal band (VDB) in the basal forebrain, suggesting that the Rab5a protein was successfully expressed in cholinergic neurons of the basal forebrain (Figure 3A,B). Enlarged Rab5a‐positive early endosomes were observed under a higher magnification fluorescence microscope (Figure 3C).

### 3.3 EA stimulation improved learning and memory by downregulating Rab5a activity in the early stage of 5 × FAD mice

**FIGURE 4:**
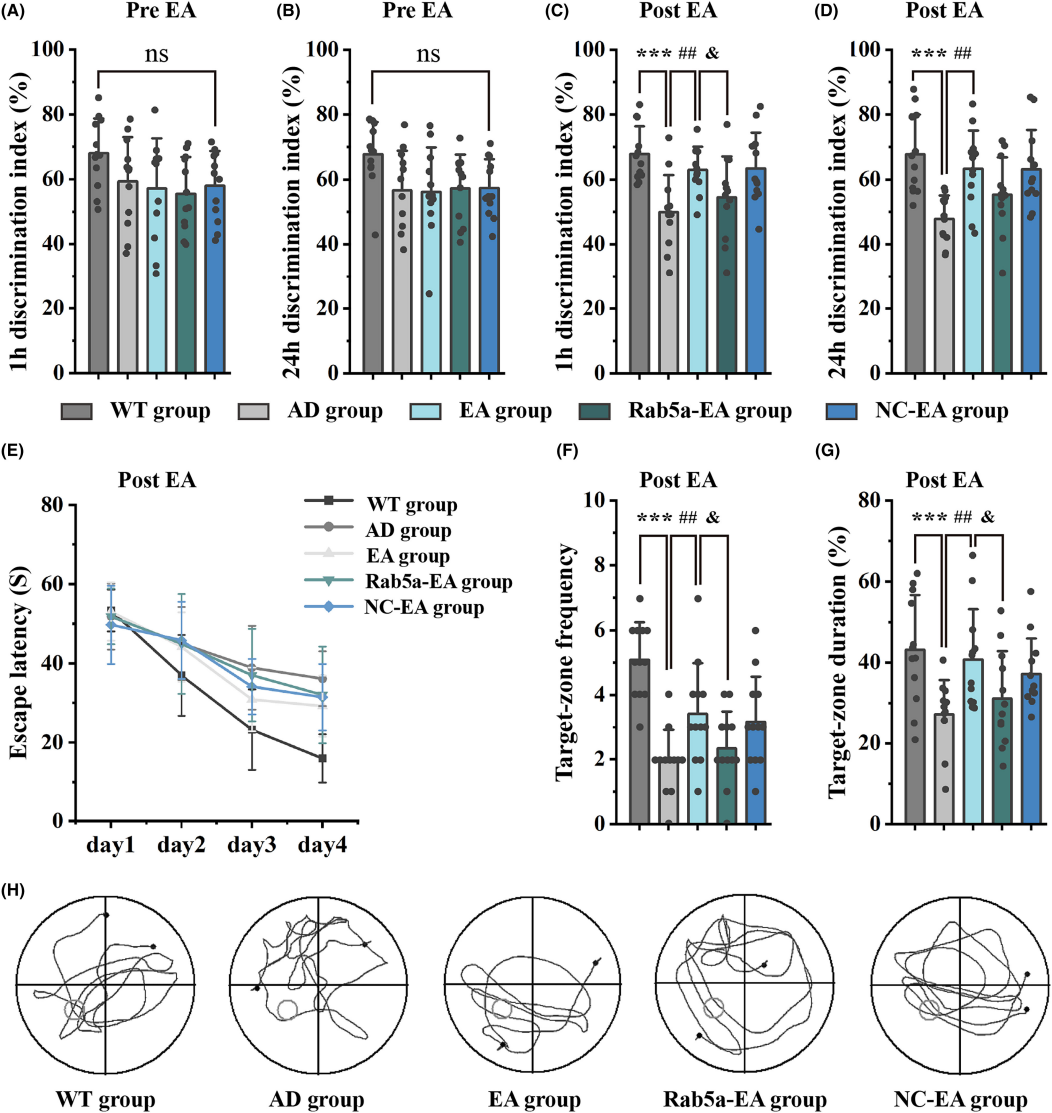
Learning and memory ability of each group, as measured by NOR and MWM test. (A‐D) 1 h and 24 h discrimination index in each group before (A, B) and after (C, D) EA intervention. (E‐G) Escape latency (E), Target‐zone frequency (F), and Target‐zone duration (G) of each group in the MWM test after EA intervention. (H) The representative movement track of each group in the MWM test (*n* = 12 for each group, ***p < 0.001 vs WT group, ##*p* < 0.01 vs AD group, ^&^ *p* < 0.05 vs EA group).

In a previous study, we found that EA at DU20 and DU24 acupoints could enhance cholinergic function and improve cognitive function in the early stage of 5 × FAD mice.^23^ In this study, we evaluated whether the EA stimulation effect against AD progression was related to the regulation of the Rab5a protein. The NOR and MWM experiments were used to evaluate object recognition memory and spatial learning memory, respectively. Object recognition memory was first detected by administering NOR tests. Before EA stimulation, there was no significant difference in the discrimination index between the WT, AD, EA, Rab5a‐EA, and NC‐EA groups (Figure 4A,B). After 28 days of EA stimulation, mice in the EA group exhibited a significantly higher discrimination index compared to the AD group. We found that the discrimination index in the Rab5a‐EA group decreased compared to the EA group (Figure 4C,D). For positioning navigation training in the MWM test, the escape latency of each group decreased as the number of training days increased (Figure 4E). In the space exploration phase, the mice in the EA group had a higher number of crossings over the platform location and spent more time exploring the target quadrant compared to the AD group. The number of crossings over the platform location and the time exploring the target quadrant in the Rab5a‐EA group decreased compared to the EA group (Figure 4F–H).

### 3.4 EA stimulation enhanced synaptic plasticity by downregulating Rab5a activity in the early stage of 5 × FAD mice

**FIGURE 5:**
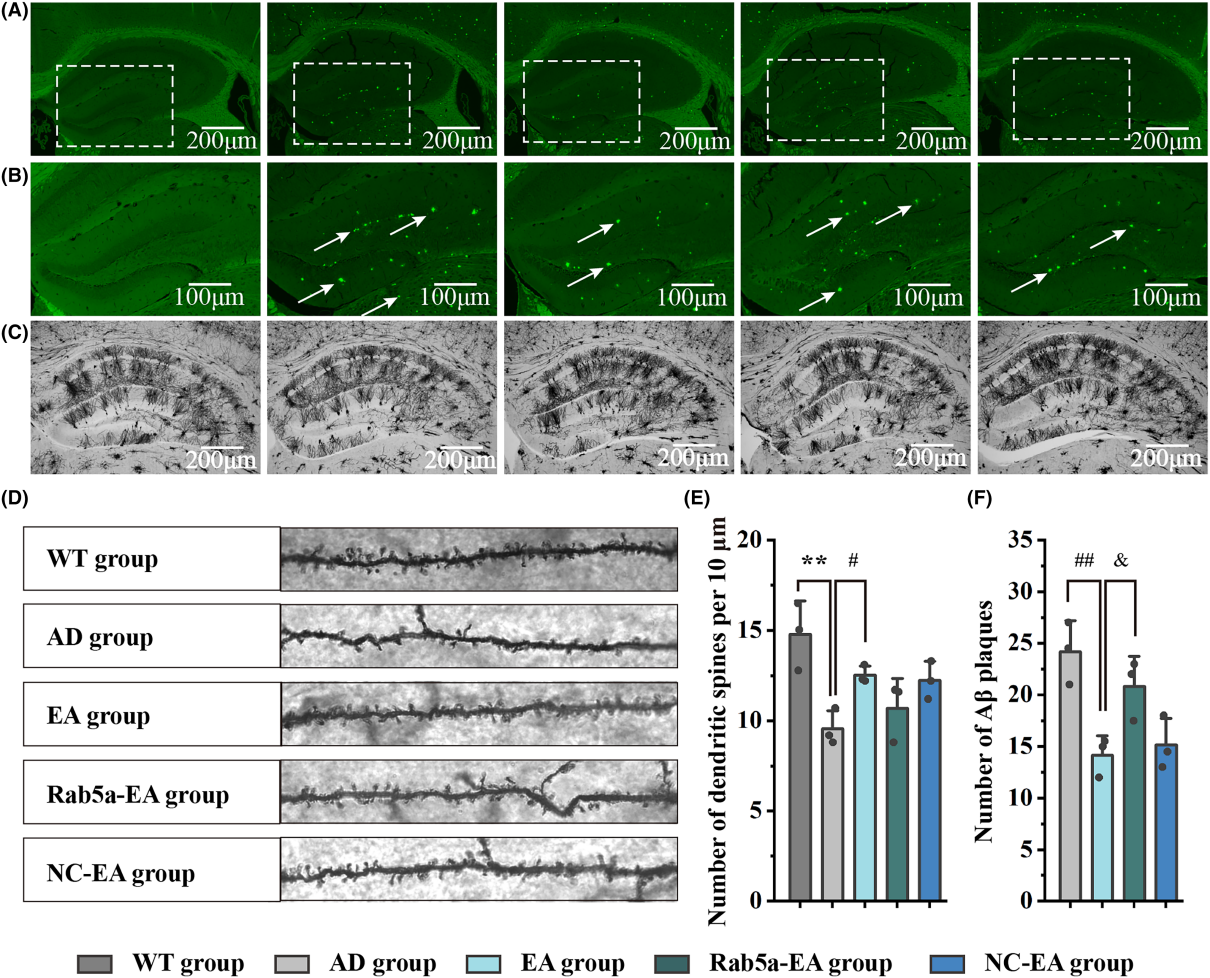
Aβ plaque deposition detected by Ths‐staining and synaptic plasticity measured by Golgi staining. (A) Representative images of Aβ plaque deposition of each group in the hippocampus after EA intervention (50×). (B) Partially enlarged view of figure A (100×). The Aβ plaques were detected in the hippocampus (as shown by the arrows). (C) Representative images of Golgi staining of each group in the hippocampus after EA intervention (50×). (D) Partially enlarged view of figure C (600×). The dendritic spines of each group are visible in the hippocampus. (E) The statistical results of the dendritic spines of each group in the hippocampus after EA intervention (*n* = 3 for each group, for each mouse, 5 dendrites were measured, and the average was taken, **p < 0.01 vs WT group, #*p* < 0.05 vs AD group). (F) The statistical results of Aβ plaques of each group in the hippocampus after EA intervention. (*n* = 3 for each group, ##*p* < 0.01 vs AD group, ^&^ *p* < 0.05 vs EA group).

The results of Thioflavin staining demonstrated that EA stimulation could decrease Aβ plaque deposition in the hippocampus and that Rab5a overexpression reversed this effect in the early stage of 5 × FAD mice (Figure 5A,B,F). Golgi staining is one of the best methods that can reflect synaptic plasticity by observing the construction of dendritic spines. Under a light microscope, we observed that the neurons in the hippocampus of each group were neatly arranged and that Golgi staining was clear and even (Figure 5C). We then observed dendritic spines at higher magnification. As the results showed, 5 × FAD mice exhibited a significantly reduced number of dendritic spines in hippocampal neurons. Twenty‐eight days of EA stimulation ameliorated the loss of dendritic spines in the hippocampus of 5 × FAD mice (Figure 5D,E).

### 3.5 EA stimulation increased the expression of cholinergic functional proteins by downregulating Rab5a activity in the early stage of 5 × FAD mice

**FIGURE 6:**
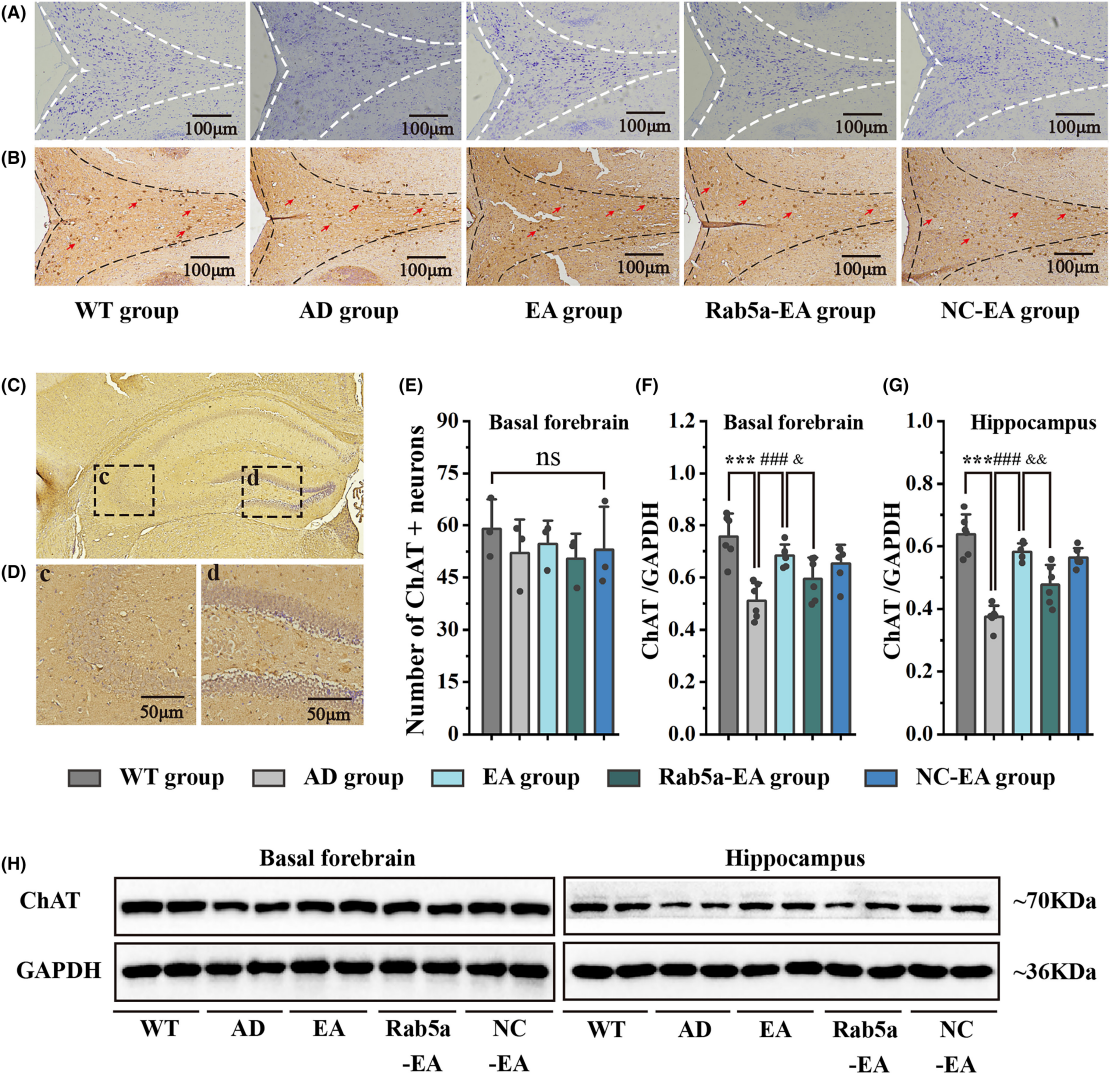
The function of cholinergic neurons detected by Nissl staining, ChAT staining, and WB. (A) Representative images of Nissl staining of each group in basal forebrain after EA intervention. (B) Representative ChAT‐antibody immunohistochemical images of each group in the basal forebrain after EA intervention (100×). Quantitative analysis was shown in the lower panel (*n* = 3 for each group). (C) Representative ChAT‐antibody immunohistochemical images of each group in the hippocampus in wild‐type mice (50×). (D) Partially enlarged view of figure C (200×). The hippocampus, including the regions of CA3 (c) and DG (d), were main targets for the projection of cholinergic neurons. (E) The statistical results of ChAT^+^ neurons of each group in the basal forebrain after EA intervention. (F‐H) ChAT protein expression levels in each group in the basal forebrain (F, H) and hippocampus (G, H) after EA intervention (n = 6 for each group, ***p < 0.001 vs WT group, ###*p* < 0.001 vs AD group, &*p* < 0.05/&&*p* < 0.01 vs EA group). ChAT, choline acetyltransferase.

**FIGURE 7:**
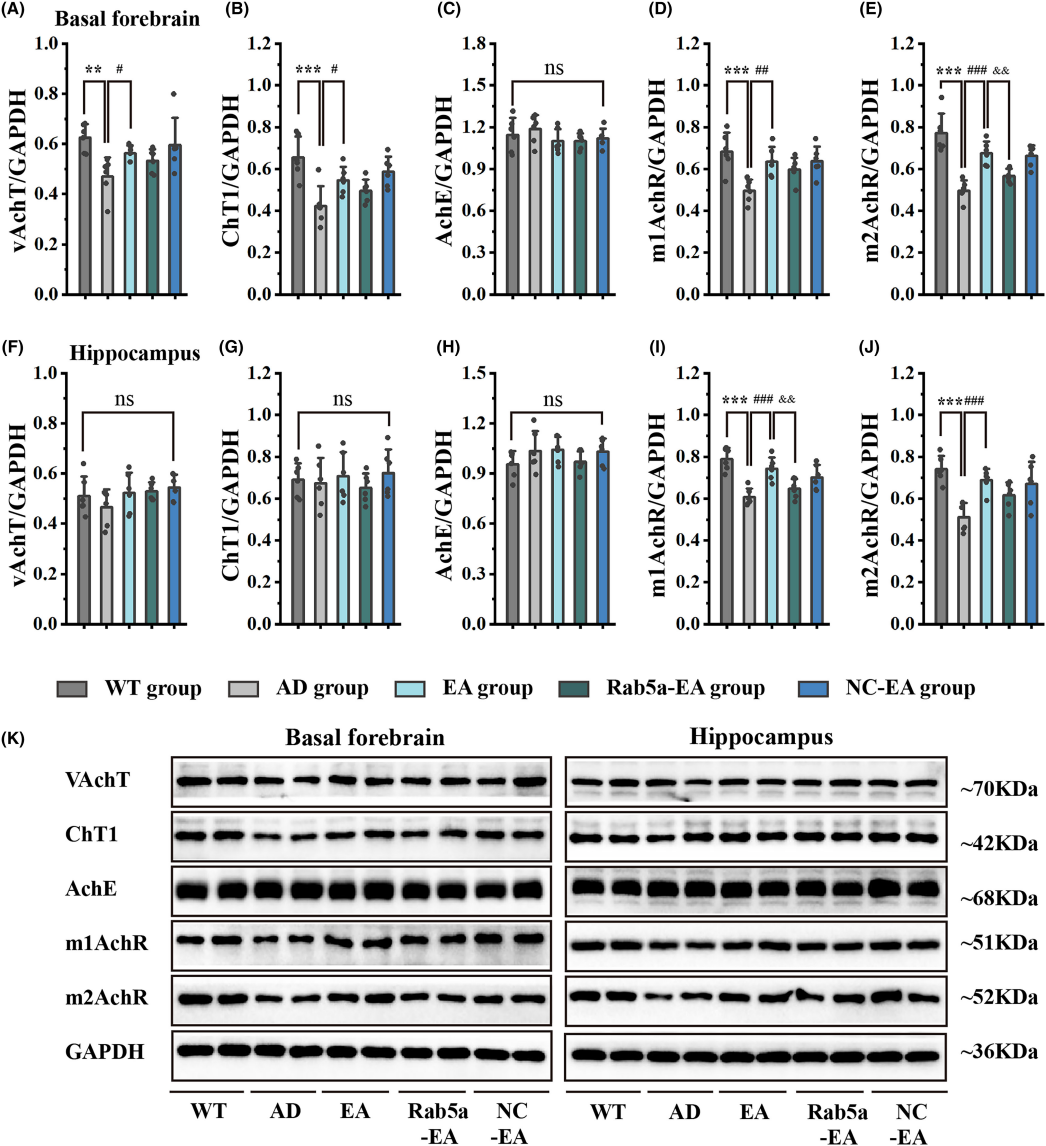
The expression levels of cholinergic‐related proteins in the basal forebrain and hippocampus. (A–E) The expression levels of vAchT (A), ChT1 (B), AchE (C), m1AchR (D), and m2AchR (E) in the basal forebrain after EA intervention. (F‐J) The expression levels of vAchT (F), ChT1 (G), AchE (H), m1AchR (I), and m2AchR (J) in the hippocampus after EA intervention (*n* = 6 for each group, **p < 0.01/***p < 0.001 vs WT group, #*p* < 0.05/##*p* < 0.01/###*p* < 0.001 vs AD group, &&*p* < 0.01 vs EA group). (K) Greyscale image of proteins. vAchT, vesicular acetylcholine transporter; ChT1, Choline transporter; AchE, enzyme acetylcholinesterase; m1AchR, Type‐1 muscarinic Acetylcholine receptor; m2AchR, Type‐2 muscarinic Acetylcholine receptor.

Nissl staining was performed to assess neuronal arrangement in the basal forebrain and demonstrated that neurons in the basal forebrain were damaged, and were irregularly arranged in the early stage of 5 × FAD mice. After 28 days of EA stimulation, the disordered arrangement and abnormal morphology of neurons in the EA group were alleviated compared to the AD group (Figure 6A). To assess the distribution of cholinergic neurons, we next immunolabeled brain sections with the anti‐ChAT antibody, showing that ChAT‐positive cholinergic neurons were widely distributed in the basal forebrain and that the hippocampus was one of the main targets for the projection of cholinergic neurons (Figure 6B–D). The statistical results demonstrated that the cholinergic neurons in the basal forebrain in 5‐month‐old 5 × FAD mice exhibited no substantial alterations compared to age‐matched wild‐type mice (Figure 6E). We next determined the ChAT protein expression in the basal forebrain and hippocampus by WB analysis. There was a significant decrease in ChAT protein expression in 5 × FAD mice, and 28 days of EA stimulation significantly up‐regulated ChAT protein expression. However, Rab5a overexpression reversed the effects of EA stimulation (Figure 6F–H). The protein levels of vAchT, ChT1, m1AchR, and m2AchR in the basal forebrain and m1AchR and m2AchR in the hippocampus significantly decreased in 5 × FAD mice compared with age‐matched wild‐type mice, and 28 days of EA stimulation significantly up‐regulated the expression of cholinergic functional proteins. However, Rab5a overexpression reversed the effects of EA stimulation. The AchE protein in the basal forebrain and AchE, vAchT, and ChT1 proteins in the hippocampus in 5‐month‐old 5 × FAD mice exhibited no substantial alterations compared with age‐matched wild‐type mice (Figure 7).

### 3.6 EA stimulation increased expression of NGF/TrkA signal‐related proteins by downregulating Rab5a activity in the early stage of 5 × FAD mice

**FIGURE 8:**
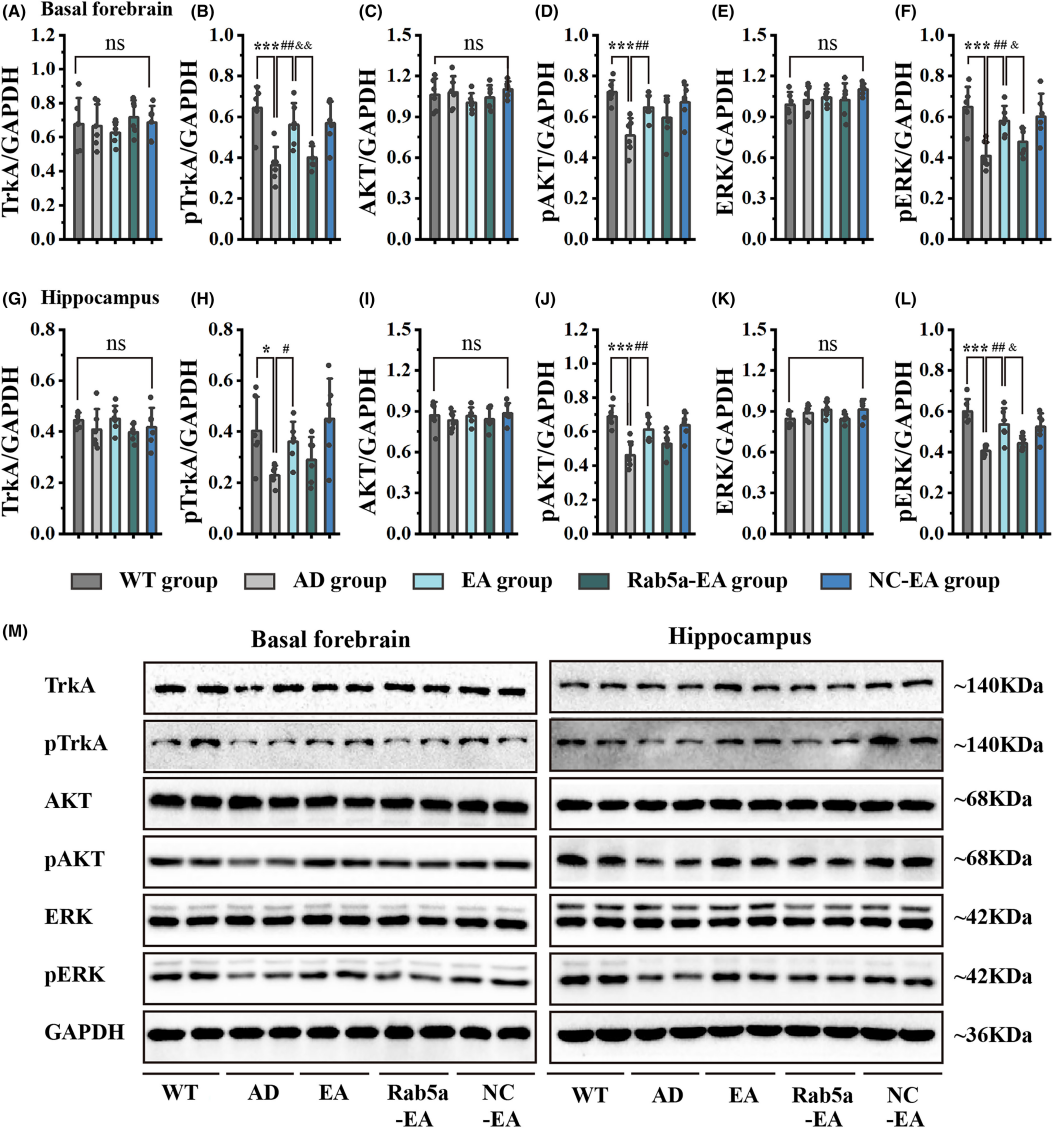
The expression levels of NGF‐related proteins in the basal forebrain and hippocampus. (A‐F) The expression levels of TrkA (A), pTrkA (B), AKT (C), pAKT (D), ERK (E), and pERK (F) in the basal forebrain after EA intervention. (G‐L) The expression levels of TrkA (G), pTrkA (H), AKT (I), pAKT (J), ERK (K), and pERK (L) in the hippocampus after EA intervention (*n* = 6 for each group, *p < 0.05/***p < 0.001 vs WT group, #*p* < 0.05/##*p* < 0.01 vs AD group, &*p* < 0.05/&&*p* < 0.01 vs EA group). (M) Greyscale image of proteins.

NGF plays an important role in maintaining the normal function of the cholinergic system. To further elucidate NGF/TrkA signaling deficits in the early stage of 5 × FAD mice, we examined the protein expression of NGF/TrkA signal transduction downstream targets in the basal forebrain and hippocampus by WB analysis. We confirmed the reduction of pTrkA levels in 5‐month‐old 5 × FAD mice relative to their age‐matched wild‐type mice in the basal forebrain and hippocampus. Similar to the lower levels of pTrkA, pAKT, and pERK also decreased in 5 × FAD mice in the basal forebrain and hippocampus. We then assessed the effects of EA stimulation in 5 × FAD mice. We detected elevated pTrkA, pAKT, and pERK levels in the basal forebrain and hippocampus after 28 days of EA stimulation compared with 5 × FAD mice. However, Rab5a overexpression reversed the effects of EA stimulation. The TrkA, AKT, and ERK proteins in the basal forebrain and hippocampus in 5‐month‐old 5 × FAD mice exhibited no substantial alterations compared with age‐matched wild‐type mice (Figure 8).

### 3.7 EA stimulation decreased Rab5a‐GTP expression in the early stage of 5 × FAD mice

**FIGURE 9:**
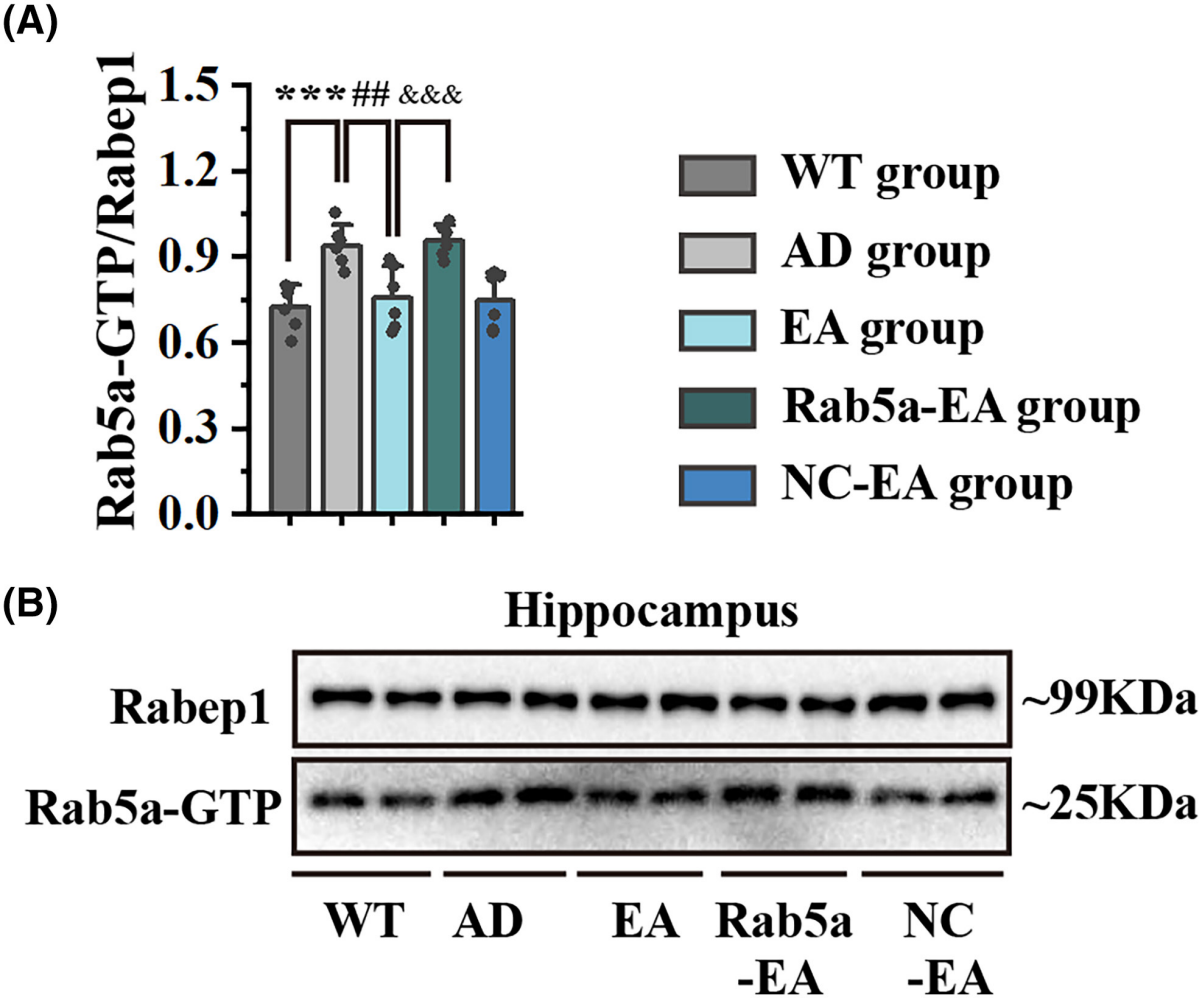
The expression levels of Rab5a‐GTP in the hippocampus. (A) The expression levels of Rab5a‐GTP in the hippocampus after EA intervention (*n* = 6 for each group, ***p < 0.001 vs WT group, ##*p* < 0.01 vs AD group, &&&*p* < 0.001 vs EA group). (B) Greyscale image of proteins.

These findings suggested that Rab5a overexpression reversed the effects of EA stimulation, including effects on cognitive function, synaptic plasticity, cholinergic function, and NGF signal transduction. To further verify our findings, WB analysis was performed to investigate the effects of EA stimulation on Rab5a‐GTP expression levels. We first confirmed the hyperactivation of Rab5a in 5 × FAD mice in the hippocampus compared to their age‐matched wild‐type mice. After 28 days of EA stimulation, the expression level of Rab5a‐GTP in the EA group decreased compared with the AD group. We also confirmed that Rab5a overexpression reversed the effects of EA stimulation, increasing Rab5a‐GTP levels in the Rab5a‐EA group compared with the EA group (Figure 9).

## 4 DISCUSSION

The neuropathological hallmark of AD has several main features, including the formation of extracellular Aβ‐containing amyloid plaque deposition and intracellular neurofibrillary tangles formed by hyperphosphorylated Tau.^25^ However, recent clinical trials targeting Aβ plaques and hyperphosphorylated Tau in AD have yet to demonstrate their efficacy.^26^ Despite early‐onset AD (EOAD) being caused by mutations in the APP, PS1, or PS2 genes, only ~1% of AD patients are EOAD, and the vast majority (~99%) of AD cases are late‐onset sporadic AD (LOAD), which has far more complex causes.^27^ Recently, GWAS and GWAMA research identified several new AD genetic risk factors and found that approximately one‐third of the risk genes encode proteins that predominantly function in the endosome‐lysosome pathway, indicating that endosome‐related gene mutations have a significant impact on AD disease progression.^12^, ^13^, ^28^ Rab5‐positive early endosome dysfunction occurs early in AD,^16^ and Rab5 hyperactivation in transgenic mice recapitulate the primary features of AD, such as early endosome enlargement, hippocampal synaptic plasticity impairment, hyperphosphorylation of the Tau protein, and cholinergic neurodegeneration.^24^ In this study, we longitudinally assessed the changes of Rab5a in AD and explored the mechanism of how Rab5a affects AD progression.

Rab5 is a critical regulator of the endosome‐lysosome pathway, which is involved in regulating early endosomes to fuse or mature into late endosomes. The Rab5 family has three members with highly similar structures and functions: Rab5a, Rab5b, and Rab5c. As an important isoform of Rab5, Rab5a was first discovered and found to be involved in the regulation of early endosome fusion.^29^ In this study, we found that Rab5a mRNA in the basal forebrain, hippocampus, and prefrontal cortex did not present significant alterations in 2‐, 3‐, 5‐ and 9‐month‐old 5 × FAD mice compared with age‐matched wild‐type mice. The unaltered Rab5a protein levels were confirmed in the hippocampus in 5‐month‐old 5 × FAD mice. However, differential expression of the total Rab5 protein was detected in AD in previous studies. Several studies have found that the Rab5 protein was selectively upregulated within the basal forebrain, frontal cortex, and hippocampus in MCI and AD patients, which was also correlated with Braak neurofibrillary tangle staging and the extent of cognitive decline.^17^, ^18^, ^30^ Studies in both APP/PS1 mice and AD‐like human iPSC supported these clinical findings.^19^, ^20^ There are two possible reasons for these differences. The first is that in different studies, Rab5 expression levels were obtained from different biological subjects (humans, APP/PS1 mice, 5 × FAD mice, and human iPSC). The second is that we examined Rab5a protein levels but not the total Rab5 protein, as in other experiments. This difference highlights the importance of investigating different Rab5 isoforms in AD. It has been demonstrated that activated Rab5a proteins (Rab5a‐GTP) recruit several effectors on the endosomal membrane to promote the fusion of endocytic vesicles with early endosomes, such as early endosome antigen 1 (EEA1) and APPL1 (adaptor protein containing PH domain, PTB domain and leucine zipper motif‐1).^14^ Rabep1 is another effector recruited by Rab5 on the membrane in a GTP‐dependent manner.^31^ In this study, Rab5‐GTP was purified through immunoprecipitation with the Rabep1 antibody. The binding rate of Rab5a to Rabep1 increased, suggesting hyperactivation of the Rab5a protein in 5 × FAD mice. Simultaneously, we also found the Rab5a‐positive early endosome enlargement from morphological observations. Therefore, we speculated that dysregulation of the endocytic pathways in the early stage of AD is caused by Rab5a hyperactivation but not Rab5a overexpression.

Cholinergic projections from the basal forebrain to the hippocampus play an important role in learning and memory processes. Changes in cholinergic function‐related proteins, degeneration of cholinergic inputs, and loss of cholinergic neurons are all pathological signs of AD. Following the cholinergic hypothesis that was first put forward in 1982, the involvement of the cholinergic system in the pathogenesis of AD has been extensively studied.^1^ Structural magnetic resonance imaging (sMRI) and Diffusion Tensor Imaging (DTI) detected atrophy of the basal forebrain and degeneration of cholinergic nerve fibers in AD patients.^32^, ^33^, ^34^ Using post‐mortem MRI and histopathology, a recent study detected volume decrease and microstructural integrity degeneration in the nucleus basalis of Meynert (NBM) of AD patients, and the decreased BFCN density was associated with reduced cholinergic tracts to its projection cerebral areas.^35^ Additionally, the degeneration and loss of BFCNs have been described in some animal models of AD.^36^, ^37^, ^38^, ^39^ The hippocampus primarily receives dense cholinergic projections from the MS and VDB nuclei of the basal forebrain, forming the basal forebrain hippocampal circuit.^40^ Studies found that cholinergic neurons in the MS and VDB nuclei play a central role in the generation of theta‐band oscillations in the hippocampus during the process of exploration, novelty detection, and memory encoding through two anatomically and functionally distinct pathways, one is the direct MS/VDB‐hippocampal cholinergic projection and the other one is the recruitment of noncholinergic neurons within the MS and VDB nuclei.^41^ In addition, MS/VDB‐hippocampal circuit acetylcholine (Ach) release regulates learning and memory processes by generating GABAergic inhibition of hippocampal dentate granule cells via astrocyte intermediaries.^42^ As stated above, this MS/VDB‐hippocampal circuit plays an important modulatory role in learning and memory and is the focus of this study.

NGF is a target‐derived neurotrophic factor needed for the phenotypic and functional maintenance of BFCNs.^43^, ^44^ Since BFCNs are highly polarized cells with long axons, signal endosomes containing NGF/TrkA complexes must control the cellular processes that occur far from the cell body, ensuring the successful formation of the NGF signal. Mounting evidence suggests that the disturbance of Rab5‐mediated endocytic pathways could play a critical role in the degeneration and loss of BFCNs in early AD stages by downregulating the NGF signal.^13^ When NGF binds to TrkA and induces its endocytosis in the axon terminals of BFCNs, TrkA‐associated RabGAP5 inactivates Rab5 by promoting its GTP hydrolysis, such that the NGF/TrkA complexes containing endocytic vesicles have low Rab5 activity and fewer changes to mature into late endosomes and enter the lysosomal system. As a result, the NGF/TrkA complexes containing vesicles exist as signaling endosomes, leading to neurite outgrowth and differentiation.^10^ RabGEF1, also known as Rabex‐5, is one member of the Rab5 GEF family. Studies have demonstrated that Rabex‐5 plays a negative regulatory role in NGF‐induced neurite outgrowth and that the knockdown of Rabex‐5 can promote NGF‐mediated biological effects in PC12 cells.^45^ Therefore, Rab5a hyperactivation in 5 × FAD mice could cause NGF/TrkA complexes containing signal endosome degradation, resulting in NGF signal loss and basal forebrain neuronal degeneration and death.

**FIGURE 10:**
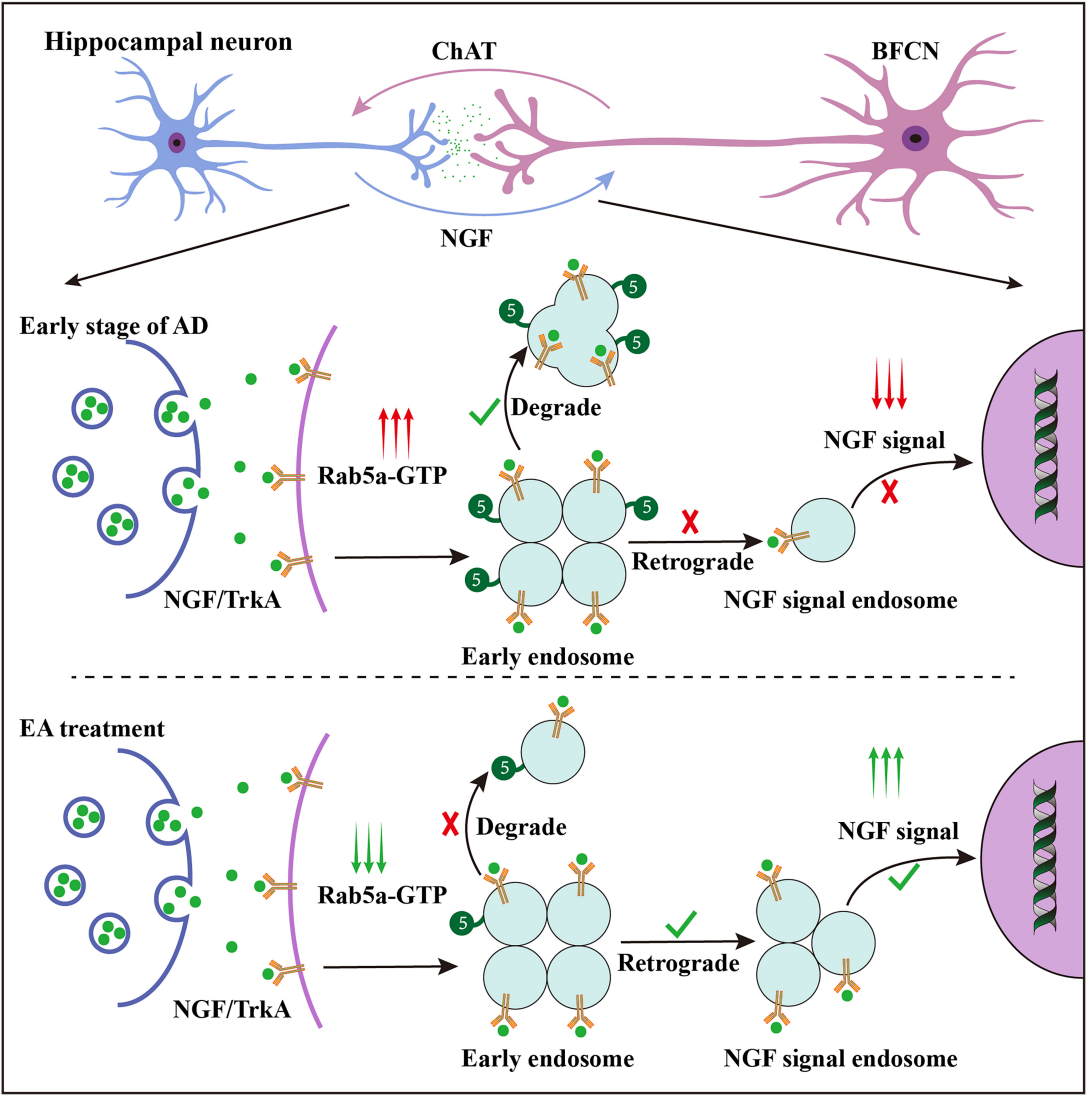
A diagram illustrating the mechanism of basal forebrain hippocampal circuit plasticity enhancement by EA treatment. Overexpression of Rab5a‐GTP in 5 × FAD mice promote early endosome fusion and facilitates NGF/TrkA complexes containing endosome degradation, resulting in NGF signal loss and a decrease in basal forebrain hippocampal circuit plasticity. EA treatment reduces the level of Rab5a‐GTP in 5 × FAD mice, increasing the NGF signal and basal forebrain hippocampal circuit plasticity in the early stage of AD.

Electroacupuncture is a form of acupuncture therapy that is a supplementary or alternative treatment for various diseases, including AD. Our previous study revealed that EA at DU20 and DU24 acupoints could ameliorate cognitive dysfunction in AD model mice.^23^, ^46^ EA treatment could inhibit hippocampal Aβ deposition and abnormal phosphorylation of the tau protein, and enhance glucose metabolism in AD model mice.^47^, ^48^ Additionally, several studies have reported that EA treatment exerts its therapeutic effects on cognition by regulating basal forebrain hippocampal circuit physiology.^22^, ^49^, ^50^ Some studies found that EA‐mediated basal forebrain hippocampal circuit plasticity enhancement is accompanied by an increase in NGF signal.^22^ In this study, we found that EA stimulation could increase the levels of phosphorylated TrkA, AKT, and ERK in the basal forebrain hippocampal circuit in the early stage of 5 × FAD mice. Considering the role of Rab5a in NGF signal transduction, Rab5a could represent a novel therapeutic target for alleviating BFCN degeneration in AD. A recent study demonstrated that EA treatment could regulate the autophagy‐lysosomal pathway to ameliorate beta‐amyloid pathology and cognitive impairment in 5 × FAD mice.^51^ This study used CRE/LOXP gene editing technology to overexpress Rab5a in the basal forebrain and revealed that 28 days of EA stimulation at DU20 and DU24 acupoints could improve learning and memory ability, increase NGF/TrkA‐mediated signaling, and enhance basal forebrain hippocampal circuit plasticity by downregulating Rab5a activity in the early stage of 5 × FAD mice (Figure 10). These findings provide further insights into possible mechanisms behind how Rab5a hyperactivation‐mediated NGF signal loss affects AD pathology and reveal the molecular mechanism of EA treatment, which increases basal forebrain hippocampal circuit plasticity and improves cognitive performance in patients with Alzheimer's disease.

There are several limitations in this study. First, only male mice were selected for study in our experiments. Alterations in sex hormones, such as progesterone and estrogen, may potentially associated with changes in brain structure and function, affecting the progression of AD.^52^ The primary risk factors of AD are age and gender,^53^ it may not be appropriate to exclude female mice in animal studies. In addition to that, it would be interesting to study the effects of EA stimulation on female mice in order to understand the gender differences in early AD EA stimulation effects (if any). Second, we mainly focused on the hippocampus, and there was insufficient detection of pathological changes in the basal forebrain. Third, although Golgi staining was used to evaluate synaptic plasticity in our present experiments, long‐term potentiation recorded by electrophysiology remains the gold standard for synaptic plasticity. Finally, we only preliminarily found that EA stimulation improves cognitive function in the early stage of AD by downregulating Rab5a activation level. In our future research, we will further explore the underlying molecular mechanism of Rab5a overactivation in the early stage of AD, and reveal with regards to how EA treatment can enhance hippocampal synaptic plasticity in the early stage of AD.

## 5 CONCLUSIONS

The conclusions obtained in this study can be summarized as follows. (1) Hyperactivation of Rab5a could be a potential cause of NGF signal loss in 5 × FAD mice. (2) EA could suppress Rab5a‐GTP to promote the transduction of NGF signaling and enhance the synaptic plasticity of the basal forebrain hippocampal circuit improving cognitive impairment in the early stage of 5 × FAD mice.

## AUTHOR CONTRIBUTIONS

Weilin Liu, Jianhong Li, Lidian Chen, Jing Tao, Shenghang Zhang designed this research; Jianhong Li, Minguang Yang, Yaling Dai, Xiaoqin Guo, Yanyi Ding, Xiaoling Li, Wenshan Xu performed experiments and analyzed data; Weilin Liu, Jianhong Li, Minguang Yang wrote and revised the manuscript. All authors reviewed the manuscript.

## FUNDING INFORMATION

This study was supported by grants from the National Natural Science Foundation of China (ref. 82104966), the Natural Science Foundation of Fujian Province (ref.2021J01959), the Natural Science Foundation of Fujian Province (ref.2023J05068).

## CONFLICT OF INTEREST STATEMENT

The authors declare that they have no conflicts of interest.

## Supporting information


**Data S1.** Supplementary Information.

## Data Availability

The data that support the findings of this study are available from the corresponding author upon reasonable request.

## References

[1] Bartus RT , Dean RR , Beer B , et al. The cholinergic hypothesis of geriatric memory dysfunction. Science. 1982;217(4558):408‐414. 7046051 10.1126/science.7046051

[2] Whitehouse PJ , Price DL , Struble RG , Clark AW , Coyle JT , DeLong MR . Alzheimer's disease and senile dementia: loss of neurons in the basal forebrain. Science. 1982;215(4537):1237‐1239. 7058341 10.1126/science.7058341

[3] Colovic MB , Krstic DZ , Lazarevic‐Pasti TD , Bondzic AM , Vasic VM . Acetylcholinesterase inhibitors: pharmacology and toxicology. Curr Neuropharmacol. 2013;11(3):315‐335. 24179466 10.2174/1570159X11311030006 PMC3648782

[4] Karran E , Mercken M , De Strooper B . The amyloid cascade hypothesis for Alzheimer's disease: an appraisal for the development of therapeutics. Nat Rev Drug Discov. 2011;10(9):698‐712. 21852788 10.1038/nrd3505

[5] Liu W , Li J , Yang M , et al. Chemical genetic activation of the cholinergic basal forebrain hippocampal circuit rescues memory loss in Alzheimer's disease. Alzheimers Res Ther. 2022;14(1):53. 35418161 10.1186/s13195-022-00994-w PMC9006585

[6] Wu H , Williams J , Nathans J . Complete morphologies of basal forebrain cholinergic neurons in the mouse. elife. 2014;3:e2444. 10.7554/eLife.02444 PMC4038840 24894464

[7] Mufson EJ , Counts SE , Ginsberg SD , et al. Nerve growth factor pathobiology during the progression of Alzheimer's disease. Front Neurosci. 2019;13:533. 31312116 10.3389/fnins.2019.00533 PMC6613497

[8] Hefti F . Nerve growth factor promotes survival of septal cholinergic neurons after fimbrial transections. J Neurosci. 1986;6(8):2155‐2162. 3746405 10.1523/JNEUROSCI.06-08-02155.1986 PMC6568758

[9] Johnston MV , Rutkowski JL , Wainer BH , Long JB , Mobley WC . NGF effects on developing forebrain cholinergic neurons are regionally specific. Neurochem Res. 1987;12(11):985‐994. 3683745 10.1007/BF00970927

[10] Liu J , Lamb D , Chou MM , Liu YJ , Li G . Nerve growth factor‐mediated neurite outgrowth via regulation of Rab5. Mol Biol Cell. 2007;18(4):1375‐1384. 17267689 10.1091/mbc.E06-08-0725 PMC1838971

[11] Delcroix JD , Valletta JS , Wu C , Hunt SJ , Kowal AS , Mobley WC . NGF signaling in sensory neurons: evidence that early endosomes carry NGF retrograde signals. Neuron. 2003;39(1):69‐84. 12848933 10.1016/s0896-6273(03)00397-0

[12] Karch CM , Goate AM . Alzheimer's disease risk genes and mechanisms of disease pathogenesis. Biol Psychiatry. 2015;77(1):43‐51. 24951455 10.1016/j.biopsych.2014.05.006 PMC4234692

[13] Xu W , Fang F , Ding J , Wu C . Dysregulation of Rab5‐mediated endocytic pathways in Alzheimer's disease. Traffic. 2018;19(4):253‐262. 29314494 10.1111/tra.12547 PMC5869093

[14] Langemeyer L , Frohlich F , Ungermann C . Rab GTPase function in endosome and lysosome biogenesis. Trends Cell Biol. 2018;28(11):957‐970. 30025982 10.1016/j.tcb.2018.06.007

[15] Cataldo AM , Barnett JL , Pieroni C , Nixon RA . Increased neuronal endocytosis and protease delivery to early endosomes in sporadic Alzheimer's disease: neuropathologic evidence for a mechanism of increased beta‐amyloidogenesis. J Neurosci. 1997;17(16):6142‐6151. 9236226 10.1523/JNEUROSCI.17-16-06142.1997 PMC6568334

[16] Troncoso JC , Cataldo AM , Nixon RA , et al. Neuropathology of preclinical and clinical late‐onset Alzheimer's disease. Ann Neurol. 1998;43(5):673‐676. 9585365 10.1002/ana.410430519

[17] Ginsberg SD , Mufson EJ , Counts SE , et al. Regional selectivity of rab5 and rab7 protein upregulation in mild cognitive impairment and Alzheimer's disease. J Alzheimers Dis. 2010;22(2):631‐639. 20847427 10.3233/JAD-2010-101080 PMC3031860

[18] Ginsberg SD , Alldred MJ , Counts SE , et al. Microarray analysis of hippocampal CA1 neurons implicates early endosomal dysfunction during Alzheimer's disease progression. Biol Psychiatry. 2010;68(10):885‐893. 20655510 10.1016/j.biopsych.2010.05.030 PMC2965820

[19] Ba L , Chen XH , Chen YL , et al. Distinct Rab7‐related endosomal‐Autophagic‐lysosomal dysregulation observed in cortex and hippocampus in APPswe/PSEN1dE9 mouse model of Alzheimer's disease. Chin Med J. 2017;130(24):2941‐2950. 29237927 10.4103/0366-6999.220311 PMC5742922

[20] Kwart D , Gregg A , Scheckel C , et al. A large panel of isogenic APP and PSEN1 mutant human iPSC neurons reveals shared endosomal abnormalities mediated by APP beta‐CTFs, not Abeta. Neuron. 2019;104(2):256‐270. 31416668 10.1016/j.neuron.2019.07.010

[21] Chen XQ , Salehi A , Pearn ML , et al. Targeting increased levels of APP in down syndrome: Posiphen‐mediated reductions in APP and its products reverse endosomal phenotypes in the Ts65Dn mouse model. Alzheimers Dement. 2021;17(2):271‐292. 32975365 10.1002/alz.12185 PMC7984396

[22] Protto V , Soligo M , De Stefano ME , et al. Electroacupuncture in rats normalizes the diabetes‐induced alterations in the septo‐hippocampal cholinergic system. Hippocampus. 2019;29(10):891‐904. 30870587 10.1002/hipo.23088

[23] Li L , Li J , Dai Y , et al. Electro‐acupuncture improve the early pattern separation in Alzheimer's disease mice via basal forebrain‐hippocampus cholinergic neural circuit. Front Aging Neurosci. 2021;13:770948. 35185516 10.3389/fnagi.2021.770948 PMC8847781

[24] Pensalfini A , Kim S , Subbanna S , et al. Endosomal dysfunction induced by directly Overactivating Rab5 recapitulates prodromal and neurodegenerative features of Alzheimer's disease. Cell Rep. 2020;33(8):108420. 33238112 10.1016/j.celrep.2020.108420 PMC7714675

[25] Malampati S , Song JX , Chun‐Kit TB , et al. Targeting Aggrephagy for the treatment of Alzheimer's disease. Cells. 2020;9(2):311. 32012902 10.3390/cells9020311 PMC7072705

[26] Hung SY , Fu WM . Drug candidates in clinical trials for Alzheimer's disease. J Biomed Sci. 2017;24(1):47. 28720101 10.1186/s12929-017-0355-7 PMC5516350

[27] Shinohara M , Fujioka S , Murray ME , et al. Regional distribution of synaptic markers and APP correlate with distinct clinicopathological features in sporadic and familial Alzheimer's disease. Brain. 2014;137(Pt 5):1533‐1549. 24625695 10.1093/brain/awu046 PMC3999719

[28] Shen R , Zhao X , He L , et al. Upregulation of RIN3 induces endosomal dysfunction in Alzheimer's disease. Transl Neurodegener. 2020;9(1):26. 32552912 10.1186/s40035-020-00206-1 PMC7301499

[29] Bucci C , Lutcke A , Steele‐Mortimer O , et al. Co‐operative regulation of endocytosis by three Rab5 isoforms. FEBS Lett. 1995;366(1):65‐71. 7789520 10.1016/0014-5793(95)00477-q

[30] Ginsberg SD , Mufson EJ , Alldred MJ , et al. Upregulation of select rab GTPases in cholinergic basal forebrain neurons in mild cognitive impairment and Alzheimer's disease. J Chem Neuroanat. 2011;42(2):102‐110. 21669283 10.1016/j.jchemneu.2011.05.012 PMC3163754

[31] Gournier H , Stenmark H , Rybin V , Lippé R , Zerial M . Two distinct effectors of the small GTPase Rab5 cooperate in endocytic membrane fusion. EMBO J. 1998;17(7):1930‐1940. 9524116 10.1093/emboj/17.7.1930 PMC1170539

[32] Teipel SJ , Flatz WH , Heinsen H , et al. Measurement of basal forebrain atrophy in Alzheimer's disease using MRI. Brain. 2005;128(Pt 11):2626‐2644. 16014654 10.1093/brain/awh589

[33] Grothe M , Heinsen H , Teipel S . Longitudinal measures of cholinergic forebrain atrophy in the transition from healthy aging to Alzheimer's disease. Neurobiol Aging. 2013;34(4):1210‐1220. 23158764 10.1016/j.neurobiolaging.2012.10.018 PMC4058576

[34] Teipel SJ , Meindl T , Grinberg L , et al. The cholinergic system in mild cognitive impairment and Alzheimer's disease: an in vivo MRI and DTI study. Hum Brain Mapp. 2011;32(9):1349‐1362. 20672311 10.1002/hbm.21111 PMC5899896

[35] Lin CP , Frigerio I , Boon B , et al. Structural (dys)connectivity associates with cholinergic cell density in Alzheimer's disease. Brain. 2022;145:2869‐2881. 35259207 10.1093/brain/awac093 PMC9420016

[36] Zhou LT , Zhang J , Tan L , et al. Elevated levels of miR‐144‐3p induce cholinergic degeneration by impairing the maturation of NGF in Alzheimer's disease. Front Cell Dev Biol. 2021;9:667412. 33898468 10.3389/fcell.2021.667412 PMC8063700

[37] Yan H , Pang P , Chen W , et al. The lesion analysis of cholinergic neurons in 5XFAD mouse model in the three‐dimensional level of whole brain. Mol Neurobiol. 2018;55(5):4115‐4125. 28597200 10.1007/s12035-017-0621-4

[38] Devi L , Ohno M . A combination Alzheimer's therapy targeting BACE1 and neprilysin in 5XFAD transgenic mice. Mol Brain. 2015;8:19. 25884928 10.1186/s13041-015-0110-5 PMC4397831

[39] Devi L , Ohno M . Phospho‐eIF2alpha level is important for determining abilities of BACE1 reduction to rescue cholinergic neurodegeneration and memory defects in 5XFAD mice. PLoS One. 2010;5(9):e12974. 20886088 10.1371/journal.pone.0012974 PMC2944882

[40] Li X , Yu B , Sun Q , et al. Generation of a whole‐brain atlas for the cholinergic system and mesoscopic projectome analysis of basal forebrain cholinergic neurons. Proceedings of the National Academy of Sciences. 2018;115:415‐420. 10.1073/pnas.1703601115 PMC5777024 29259118

[41] Dannenberg H , Pabst M , Braganza O , et al. Synergy of direct and indirect cholinergic septo‐hippocampal pathways coordinates firing in hippocampal networks. J Neurosci. 2015;35(22):8394‐8410. 26041909 10.1523/JNEUROSCI.4460-14.2015 PMC6605336

[42] Pabst M , Braganza O , Dannenberg H , et al. Astrocyte intermediaries of septal cholinergic modulation in the hippocampus. Neuron. 2016;90(4):853‐865. 27161528 10.1016/j.neuron.2016.04.003

[43] Mitra S , Behbahani H , Eriksdotter M . Innovative therapy for Alzheimer's disease‐with focus on biodelivery of NGF. Front Neurosci. 2019;13:38. 30804738 10.3389/fnins.2019.00038 PMC6370742

[44] Biane J , Conner JM , Tuszynski MH . Nerve growth factor is primarily produced by GABAergic neurons of the adult rat cortex. Front Cell Neurosci. 2014;8:220. 25147503 10.3389/fncel.2014.00220 PMC4124705

[45] Tam SY , Lilla JN , Chen CC , Kalesnikoff J , Tsai M . RabGEF1/Rabex‐5 regulates TrkA‐mediated neurite outgrowth and NMDA‐induced signaling activation in NGF‐differentiated PC12 cells. PLoS One. 2015;10(11):e142935. 10.1371/journal.pone.0142935 PMC4654474 26588713

[46] Li J , Zhang B , Jia W , et al. Activation of adenosine monophosphate‐activated protein kinase drives the aerobic glycolysis in hippocampus for delaying cognitive decline following Electroacupuncture treatment in APP/PS1 mice. Front Cell Neurosci. 2021;15:774569. 34867206 10.3389/fncel.2021.774569 PMC8636716

[47] Sun RQ , Wang ZD , Zhao J , et al. Improvement of electroacupuncture on APP/PS1 transgenic mice in behavioral probably due to reducing deposition of Abeta in hippocampus. Anat Rec. 2021;304(11):2521‐2530. 10.1002/ar.24737 34469051

[48] Xu A , Zeng Q , Tang Y , et al. Electroacupuncture protects cognition by regulating tau phosphorylation and glucose metabolism via the AKT/GSK3beta signaling pathway in Alzheimer's disease model mice. Front Neurosci. 2020;14:585476. 33328854 10.3389/fnins.2020.585476 PMC7714768

[49] Rocco ML , Pristera A , Pistillo L , et al. Brain cholinergic markers and tau phosphorylation are altered in experimental type 1 diabetes: normalization by electroacupuncture. J Alzheimers Dis. 2013;33(3):767‐773. 23001708 10.3233/JAD-2012-121309

[50] Soligo M , Piccinin S , Protto V , et al. Recovery of hippocampal functions and modulation of muscarinic response by electroacupuncture in young diabetic rats. Sci Rep. 2017;7(1):9077. 28831054 10.1038/s41598-017-08556-z PMC5567336

[51] Zheng X , Lin W , Jiang Y , et al. Electroacupuncture ameliorates beta‐amyloid pathology and cognitive impairment in Alzheimer disease via a novel mechanism involving activation of TFEB (transcription factor EB). Autophagy. 2021;17(11):3833‐3847. 33622188 10.1080/15548627.2021.1886720 PMC8632298

[52] Barth C , Crestol A , de Lange AG , et al. Sex steroids and the female brain across the lifespan: insights into risk of depression and Alzheimer's disease. Lancet Diabetes Endocrinol. 2023;11(12):926‐941. 37865102 10.1016/S2213-8587(23)00224-3

[53] Vina J , Lloret A . Why women have more Alzheimer's disease than men: gender and mitochondrial toxicity of amyloid‐beta peptide. J Alzheimers Dis. 2010;20(Suppl 2):S527‐S533. 20442496 10.3233/JAD-2010-100501

