# Supplementary material for: Electroacupuncture regulates Rab5a‐mediating NGF transduction to improve learning and memory ability in the early stage of AD mice
Source: CNS Neurosci Ther. 2024 May 23;30(5):e14743. doi: 10.1111/cns.14743 (PMC11112630; doi:10.1111/cns.14743)

**Full unedited gel/blot for Figure 2G**

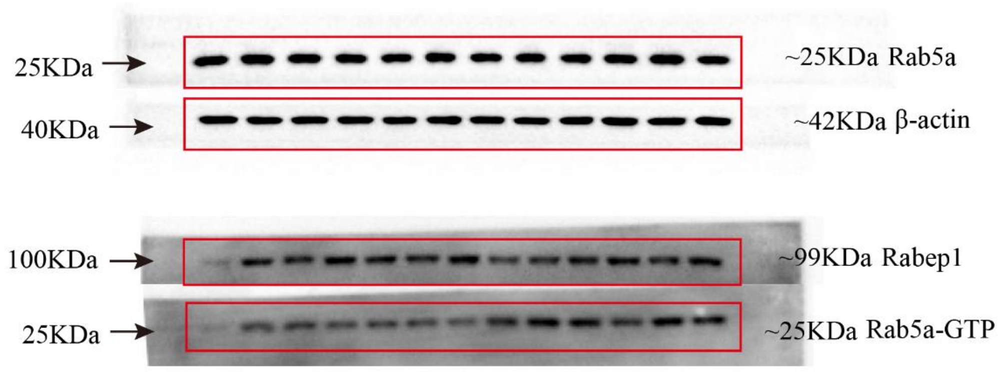


**Full unedited gel/blot for Figure 6H**

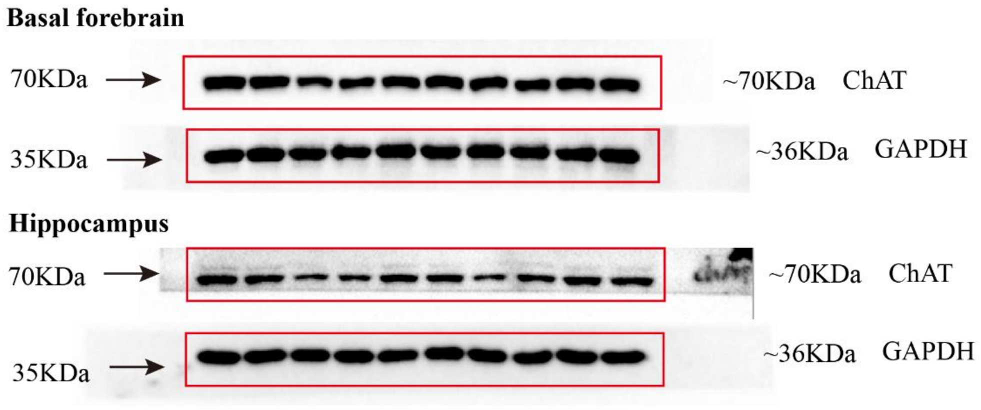

**Full unedited gel/blot for Figure 7K**

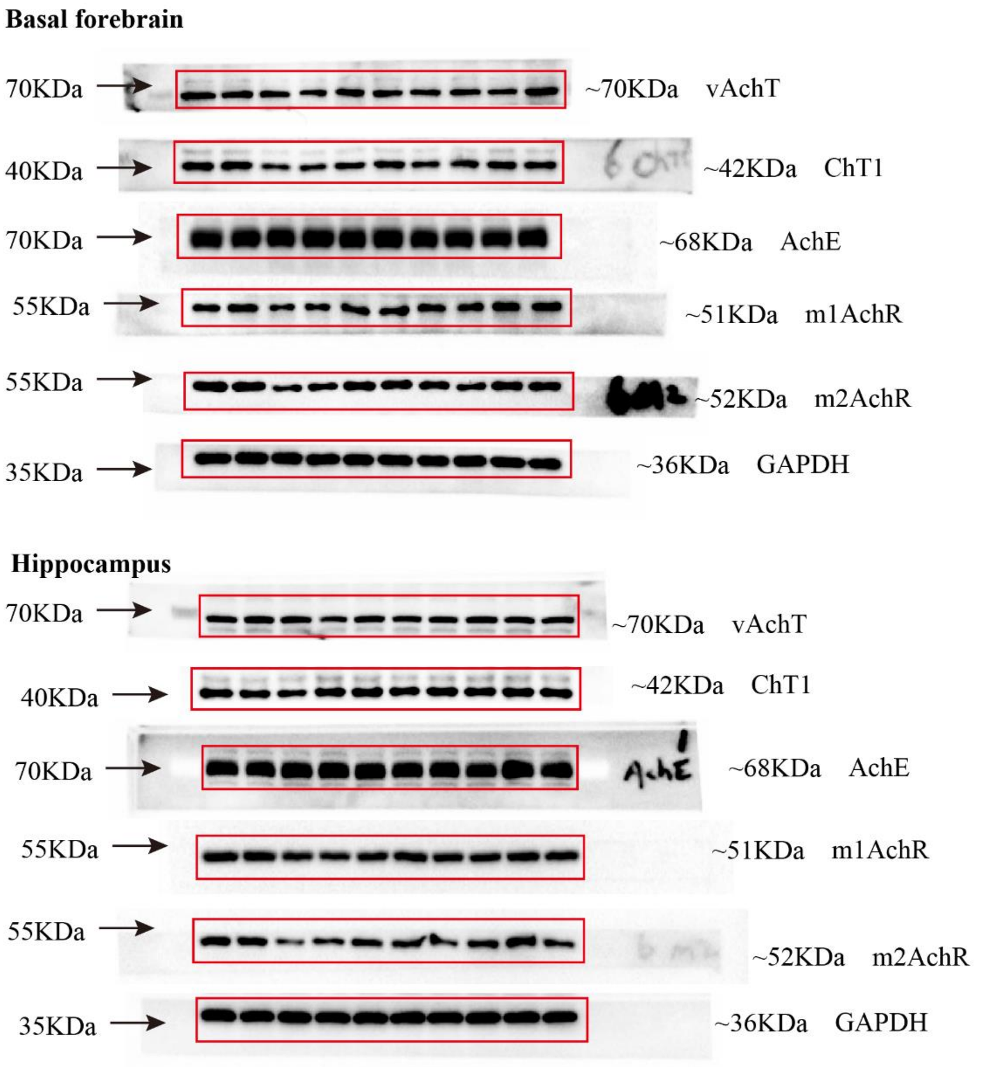

**Full unedited gel/blot for Figure 8M**

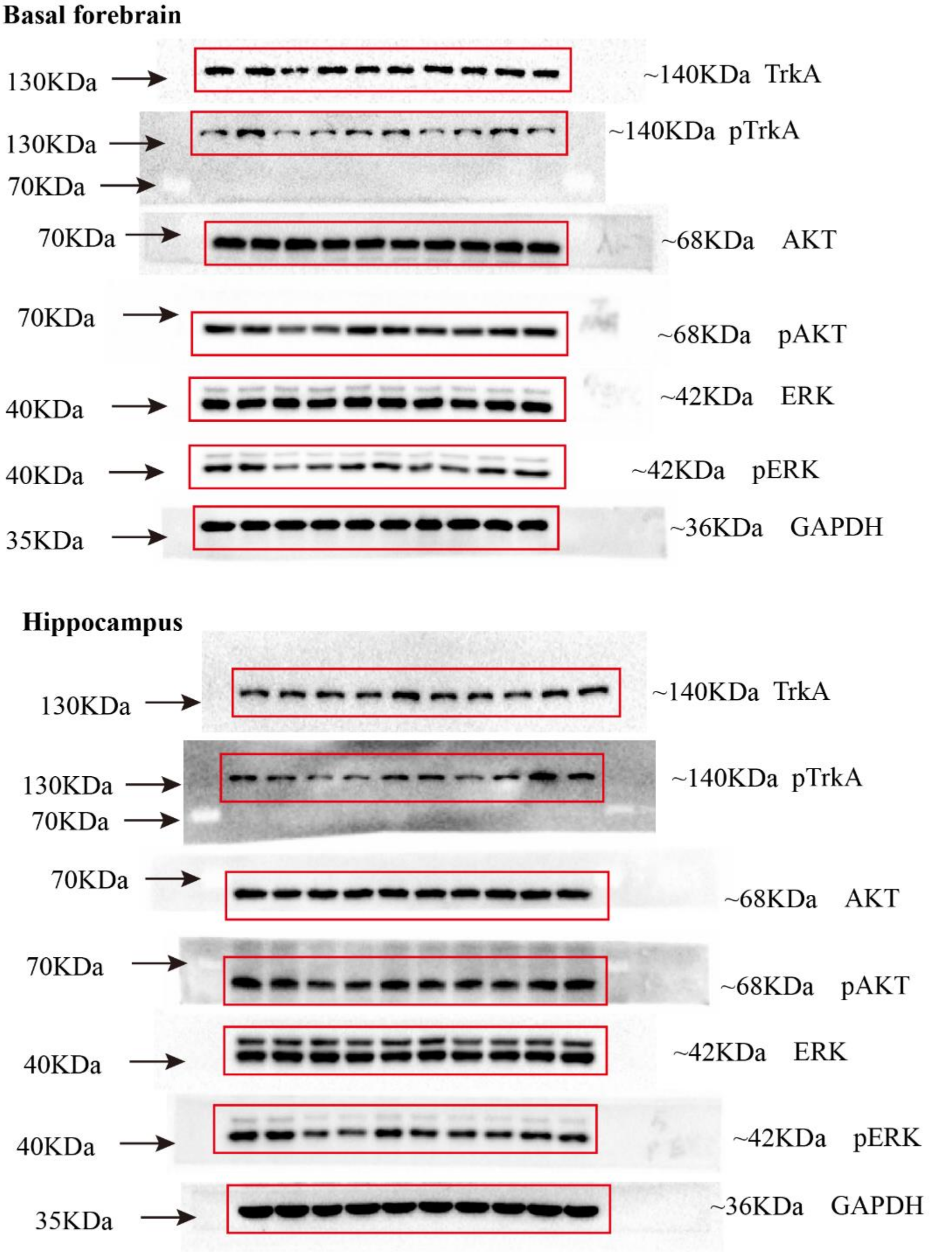


**Full unedited gel/blot for Figure 9B**

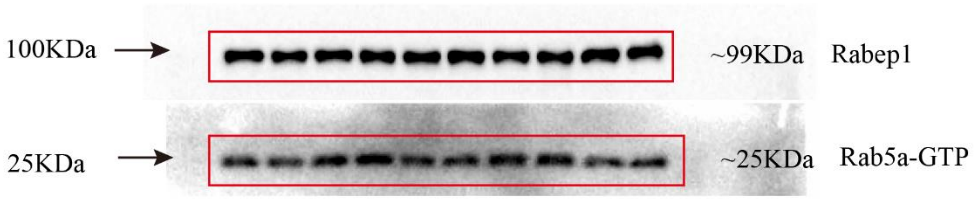

Supplement: Supplementary file 1 — Data S1. Supplementary Information. [file CNS-30-e14743-s001.pdf]
